# Chemoproteomic profiling reveals that cathepsin D off-target activity drives ocular toxicity of β-secretase inhibitors

**DOI:** 10.1038/ncomms13042

**Published:** 2016-10-11

**Authors:** Andrea M. Zuhl, Charles E. Nolan, Michael A. Brodney, Sherry Niessen, Kevin Atchison, Christopher Houle, David A. Karanian, Claude Ambroise, Jeffrey W. Brulet, Elizabeth M. Beck, Shawn D. Doran, Brian T. O'Neill, Christopher W. am Ende, Cheng Chang, Kieran F. Geoghegan, Graham M. West, Joshua C. Judkins, Xinjun Hou, David R. Riddell, Douglas S. Johnson

**Affiliations:** 1 Pfizer Worldwide Research and Development, Cambridge, Massachusetts 02139, USA; 2 Worldwide Medicinal Chemistry; 3 Neuroscience Research Unit; 4 Pfizer Worldwide Research and Development, San Diego, California 92121, USA; 5 Pfizer Worldwide Research and Development, Groton, Connecticut 06340, USA; 6 Drug Safety Research and Development; 7 Pharmacokinetics, Dynamics and Metabolism; 8 Structural Biology and Biophysics Group

## Abstract

Inhibition of β-secretase BACE1 is considered one of the most promising approaches for treating Alzheimer's disease. Several structurally distinct BACE1 inhibitors have been withdrawn from development after inducing ocular toxicity in animal models, but the target mediating this toxicity has not been identified. Here we use a clickable photoaffinity probe to identify cathepsin D (CatD) as a principal off-target of BACE1 inhibitors in human cells. We find that several BACE1 inhibitors blocked CatD activity in cells with much greater potency than that displayed in cell-free assays with purified protein. Through a series of exploratory toxicology studies, we show that quantifying CatD target engagement in cells with the probe is predictive of ocular toxicity *in vivo*. Taken together, our findings designate off-target inhibition of CatD as a principal driver of ocular toxicity for BACE1 inhibitors and more generally underscore the power of chemical proteomics for discerning mechanisms of drug action.

## Abstract (web summary)

Several β-secretase (BACE) inhibitors exhibit unexplained ocular toxicity in preclinical studies. Here the authors generate a clickable photoaffinity probe to interrogate off-targets in cells and animals, and identify inhibition of cathepsin D as a driver of ocular toxicity.

## Graphical abstract

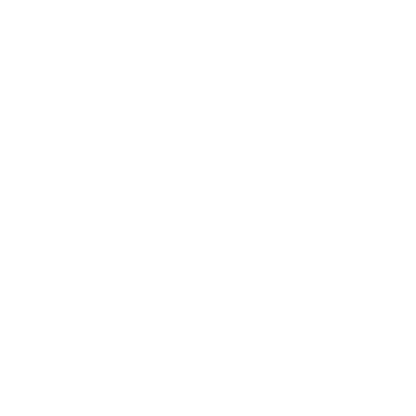

In the development of therapeutic agents or chemical probes, on-target efficacy is typically tested in several cellular models before time consuming and costly *in vivo* testing. However, in many cases selectivity is only confirmed with *in vitro* assays against related protein targets or a broad panel of representative enzymes and receptors. This approach can fail to accurately predict selectivity, as compound potency can change markedly in complex biological settings^1^,^2^ and pharmacology is often shared outside of gene families^3^,^4^,^5^. It can therefore be difficult to pinpoint whether unanticipated compound effects observed in animal models are a result of poorly understood consequences of on-target engagement, interactions with uncharacterized off-targets, or selectivity assays that do not accurately represent the *in vivo* environment. Without a specific mechanism of action, improving compound properties or defining therapeutic windows for future *in vivo* studies is substantially more challenging.

Understanding the drivers of undesired compound effects has been especially challenging for small molecule inhibitors of β-secretase BACE1 (β-site APP-cleaving enzyme 1). The amyloid cascade hypothesis of Alzheimer's disease (AD) links disease pathology to an accumulation of cerebral amyloid beta (Aβ)^6^. BACE1 initiates the production of Aβ from amyloid precursor protein (APP), and therefore blocking the activity of this enzyme is considered one of the most promising approaches for treating AD^7^,^8^. Considerable progress towards small molecule BACE1 inhibitors has been made and several inhibitors are in clinical trials, but to date none have received FDA approval^9^,^10^. Safety liabilities have been a major cause of BACE1 inhibitor attrition, and in particular, both Eli Lilly & Co. first generation clinical candidate LY2811376 (**1**) (ref. 11), and Amgen preclinical candidate AMG-8718 (**2**) (ref. 12), were withdrawn from development after exhibiting ocular toxicity in preclinical animal models. Both inhibitors were found to cause an accumulation of autofluorescent material in the retinal pigment epithelium (RPE) and subsequent retinal photoreceptor degeneration^11^,^12^, which would be expected to eventually result in severe visual impairment^13^. The RPE is a non-regenerating layer of cells that has a number of important physical and biochemical functions crucial to the visual cycle, including the daily recycling of shed autofluorescent photoreceptor outer segments (POS)^13^,^14^. The accumulated RPE autofluorescent material observed with either LY2811376 or AMG-8718 treatment is consistent with impaired phagolysosomal POS degradation. Notably, LY2811376 also induced this effect in mice lacking the BACE1 gene, suggesting off-target effects^11^.

BACE1 is a member of the pepsin aspartyl protease superfamily and blockade of enzyme function is typically achieved by active site-directed inhibitors that non-covalently engage the catalytic aspartate residues^9^,^10^. Selectivity is therefore typically evaluated against a panel of purified aspartyl proteases (for example, BACE2, cathepsin D, cathepsin E, pepsin and renin) and for the most part, inferred across the broader proteome^9^,^10^. With the exception of the closely related enzyme BACE2, selectivity against other aspartyl proteases was reported to be >60-fold for LY2811376 (ref. 11), and >1,000-fold for AMG-8718 (ref. 15). Selectivity against the endolysosomal aspartyl protease cathepsin D (CatD) was considered particularly significant as this enzyme had been annotated as a key component of the POS phagocytic pathway^16^, and CatD deficiency in mammals^17^,^18^ or in humans^19^ had been shown to cause accumulations of autofluorescent material and visual impairment. Consequently, concerns were raised as to whether inhibition of BACE1—either alone or in combination off-target effects—might play a role in ocular toxicity. BACE1 knockout (KO) mice have been described as overtly phenotypically normal^20^, although some studies report hypomyelination of peripheral nerves in neonates and delayed remyelination following peripheral nerve injury^21^,^22^. One study of BACE1 knockout mice identified an ocular pathology^23^, which in that study could also be induced *in vivo* in wild-type mice with BACE1 inhibitor IV (**3**) (ref. 24). Other studies that specifically examined the retinas of BACE1 KO mice^11^ or rats^12^ did not find any ocular irregularities. BACE2 KO mice have displayed coat colour defects^25^, but have otherwise been described as phenotypically normal^22^,^25^. Additional potential off-targets have remained largely unexplored including several components of the POS phagocytic pathway or proteins with genetic associations to accumulation of autofluorescent material^14^. After 2010 when LY2811376 was withdrawn from clinical development, patients in some BACE1 clinical trials have had to undergo regular ophthalmologic exams.

Here we use quantitative chemoproteomics to perform the first target agnostic search for the mechanism of BACE1 inhibitor ocular toxicity. We identify CatD as a principal off-target of BACE1 inhibitors in a human RPE cell line and demonstrate that several BACE1 inhibitors show substantially enhanced potency for CatD in live cells compared with cell-free assays utilizing purified proteins. We combine these cellular target engagement measurements with exploratory toxicology studies and exposure-response analyses to designate off-target inhibition of CatD as a principal driver of ocular toxicity for BACE1 inhibitors.

## Results

### BACE1 inhibitor PF-9283 induces ocular toxicity in mammals

**Figure 1:**
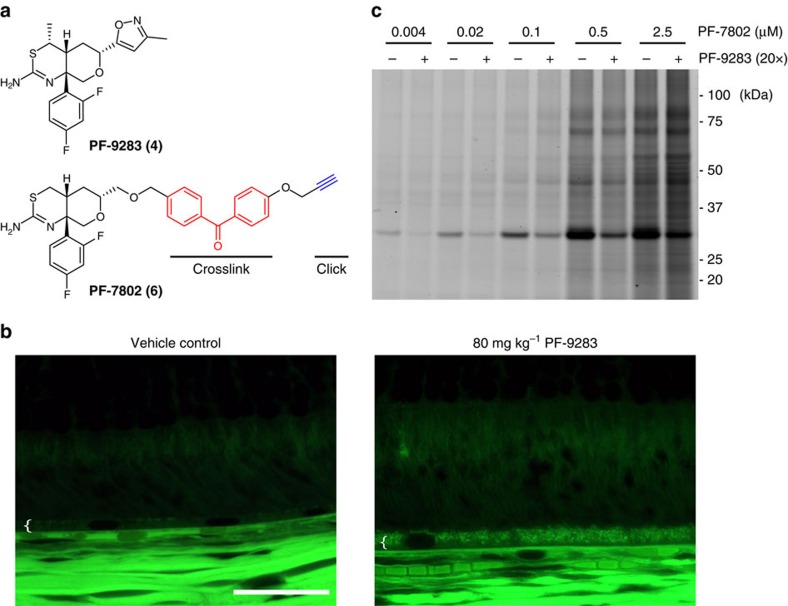
PF-7802 probe design and results in live retinal pigmented epithelial (RPE) cells. (**a**) Structure of BACE1 inhibitor PF-9283 and clickable photoprobe analog PF-7802. For a comparison of the aspartyl protease inhibitory activities of PF-9283 and PF-7802, see Supplementary Table 3. (**b**) Representative fluorescent microscopic images of the retinas of rats dosed daily with vehicle (left) or 80 mg kg^−1^ PF-9283 (right) for 14 days (*n*=5 or 6 per group). White bracket indicates the RPE layer; accumulated autofluorescent granules are visible after PF-9283 treatment. H&E stain, × 40 objective, scale bar represents 50 μm. For a summary of ocular findings in wild-type and BACE1^(−/−)^ mice, see Supplementary Table 2. (**c**) In-gel fluorescence analysis of PF-7802-labelled ARPE-19 cells. Live ARPE-19 cells were incubated with PF-7802 in the presence or absence of excess PF-9283, followed by ultraviolet irradiation, cell lysis, *in vitro* click chemistry with TAMRA-azide, and SDS–PAGE. See Supplementary Fig. 2 for scheme. Concentration-dependent labelling of a ∼30 kDa band, which was competed by a 20-fold excess of PF-9283, was observed.

We recently disclosed a novel series of reversible active site-directed thioamidine BACE1 inhibitors including isoxazole analog PF-9283 (**4**, Fig. 1a), which displayed robust central Aβ-lowering efficacy and more than 200-fold selectivity for BACE1 over cathepsins D and E in assays with purified proteins^26^ (Supplementary Table 1). On the basis of these promising attributes, we advanced PF-9283 to 14-day exploratory toxicology studies in rats. As part of these studies, we evaluated haematoxylin and eosin (H&E)-stained retinal sections from PF-9283-treated animals for ocular toxicity. Fluorescence microscopy revealed an accumulation of autofluorescent granules (AFG) in the RPE (Fig. 1b) of rats dosed daily with 80 mg kg^−1^ PF-9283. PF-9283-induced increases in AFG could be observed as early as 4 days into the study. This abnormality did not increase in severity with time (up to 30-day study duration), but did become more frequent among the cohort (Supplementary Table 2). We observed the same phenotype when PF-9283 was dosed in exon1-disrupted BACE1 knockout mice^27^, which had normal retinas after dosing with a vehicle control (Supplementary Fig. 1). In addition, the incidence of ocular toxicity was roughly equivalent between cohorts of wild-type and BACE1 knockout mice dosed with PF-9283 in 14- or 30-day dosing studies (Supplementary Table 2). Collectively, this evidence was consistent with similar studies performed with LY2811376 (ref. 11) and AMG-8718 (ref. 12), and suggested that BACE1 does not mediate ocular toxicity.

### Design of probe PF-7802 and profiling of targets in cells

If on-target BACE1 inhibition was not responsible for PF-9283 toxicity, we wondered what off-target(s) could be causative. To broadly evaluate potential off-targets, we followed an approach that has been used successfully for several other drugs^4^,^28^,^29^,^30^ and generated a chemical probe where PF-9283 was modified to incorporate a benzophenone for covalent attachment to protein targets upon ultraviolet irradiation, and an alkyne for conjugation to biotin or fluorophore reporters via a copper-catalysed azide-alkyne cycloaddition (CuAAC or ‘click' chemistry)^31^. The resulting probe PF-7802 (**6**, Fig. 1a) maintained a similar aspartyl protease pharmacological profile to PF-9283, albeit with reduced cellular potency for BACE1 (Supplementary Table 3). However, PF-7802 still substantially inhibited the generation of sAPPβ—the direct product of BACE1 cleavage of APP—in a cellular assay of BACE1 activity (half-maximum inhibitory concentration or IC_50_ value of 320 nM).

To identify the potential binding partners, we treated ARPE-19 cells, a human cell line derived from the RPE with a normal karyotype^32^, with PF-7802 (0.004–2.5 μM) for 30 min in the presence or absence of a 20-fold excess of PF-9283, and then irradiated the cells with ultraviolet light (365 nm, 15 min). After cell lysis, probe-labelled proteins were conjugated to TAMRA-azide, separated by SDS–polyacrylamide gel electrophoresis (PAGE) and visualized by in-gel fluorescence scanning (Fig. 1c, see Supplementary Fig. 2 for scheme). Proteins that are specifically labelled by PF-7802 are those that exhibit a decrease in fluorescent signal in PF-9283-pretreated samples. Equal fluorescent signals in DMSO- and PF-9283-pretreated samples indicate non-specific labelling, which can be due to the reactive nature of the photoreactive species or activity unique to PF-7802. We observed concentration-dependent labelling of a ∼30 kDa band that was competed by pretreatment with PF-9283, indicating specific labelling. This target was detected with as little as 0.004 μM PF-7802 and was the major competed target by gel-based analysis.

### Mass spectrometry analysis of PF-7802-labelled proteins

**Figure 2:**
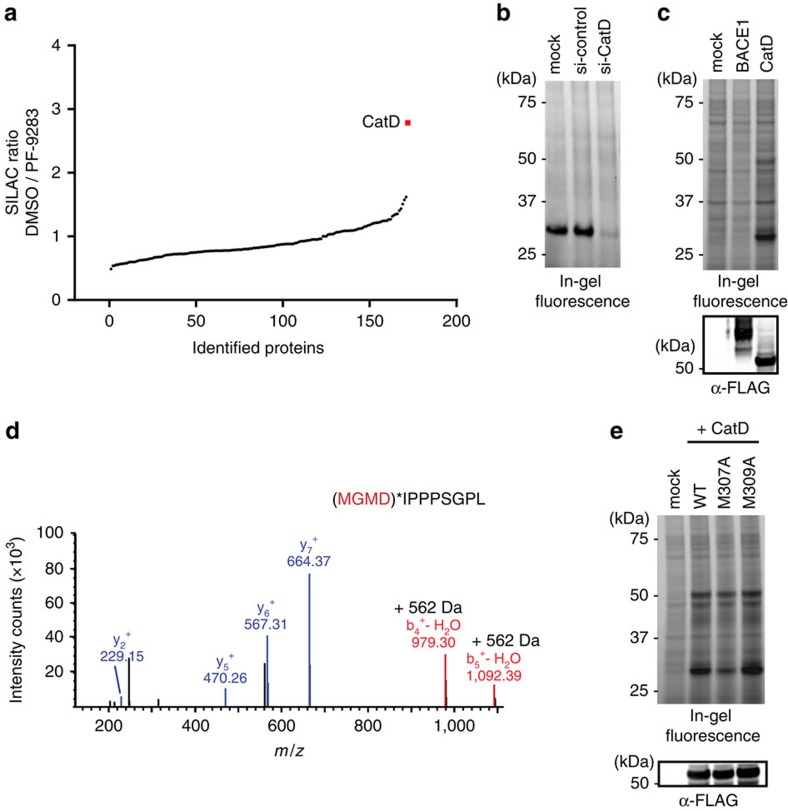
Identification of PF-7802-labelled proteins and peptides. (**a**) SILAC mass spectrometry ratios for proteins identified from live ARPE-19 cells pretreated with DMSO or 2 μM PF-9283 and photolabelled with 100 nM PF-7802, followed by click chemistry with biotin-azide, enrichment with streptavidin, and on-bead trypsin digest. Light (*n*=3) or heavy (*n*=1) cells were pretreated with PF-9283 and the corresponding control cells were treated with DMSO. SILAC ratios are the median of at least five peptides per protein identification for four independent experiments. (**b**) In-gel fluorescence analysis of ARPE-19 cells transfected with CatD siRNA (si-CatD) or control siRNA (si-control) and photolabelled with 100 nM PF-7802. Cells treated with si-CatD showed a reduction in PF-7802 labelling relative to mock and si-control transfected cells. For CatD and actin western blots see Supplementary Fig. 4. (**c**) In-gel fluorescence analysis of HEK293T cells transiently transfected with WT BACE1 or WT CatD and photolabelled with 1 μM PF-7802 (top). (**d**) MS^2^ spectrum of peptide MGMDIPPPSGPL covalently modified within the sequence MGMD by incubation of 2.2 μM purified human CatD with 2 μM PF-7802. PF-7802 only modified CatD at this sequence (indicated by + 562 Da in b_4_^+^ daughter ion *m*/*z*), which is not conserved in BACE1 (see also Supplementary Figs 6–8). (**e**) In-gel fluorescence analysis of HEK293T cells transiently transfected with WT CatD, CatD^M307A^ or CatD^M309A^, and photolabelled with 1 μM PF-7802 (top). For **c** and **e**, differences in labelling were independent of expression level as indicated by FLAG western blots (bottom). For full FLAG western blots see Supplementary Fig. 4.

We also analysed probe-labelled protein targets by quantitative mass spectrometry using stable-isotope labelling by amino acids in cell culture (SILAC)^33^. This allowed us to simultaneously identify the 30 kDa band and also detect any additional probe-labelled proteins that could not be visualized by in-gel fluorescence. Isotopically labelled ARPE-19 cells were pretreated for 30 min with 2 μM PF-9283 or DMSO, before photoaffinity labelling with 100 nM PF-7802 as before. After cell lysis, heavy and light proteomes were combined, and conjugated to biotin-azide via click chemistry^31^. Probe-labelled proteins were enriched over streptavidin, digested on-bead with trypsin, and analysed by liquid chromatography-tandem mass spectrometry (LC-MS/MS, Fig. 2a, Supplementary Data 1 and Supplementary Fig. 3). We identified 172 proteins in this analysis, almost all of which had SILAC ratios between 0.5 and 2.0, indicating either non-specific enrichment with streptavidin or non-specific labelling by PF-7802. Cathepsin D (CatD) was the only protein changed more than 2-fold by the pretreatment with PF-9283 (heavy/light or H/L=2.7). We repeated the experiment with several control compounds: LY2886721 (**5**), Eli Lilly & Co.'s second-generation clinical candidate, which does not inhibit purified CatD *in vitro* (IC_50_>100 μM)^34^; LY2811376 (**1**), which inhibits both BACE1 and CatD; and CP-108671 (**7**) (ref. 35), a pepstatin-based renin inhibitor with potent off-target CatD inhibition, but no BACE1 activity (Supplementary Fig. 3). Pretreatment with either LY2811376 or CP-108671 blocked labelling of the 30 kDa band by PF-7802 and resulted in high SILAC ratios for CatD. By contrast, LY2886721-pretreated samples were not significantly distinguishable from DMSO controls. No additional proteins identified in any of the above studies had SILAC ratios of >2.0. Together, these results showed CatD was a specific target of PF-7802 in ARPE-19 cells and that photolabelling of CatD was blocked by the direct binding of PF-9283, not as a downstream consequence of BACE1 inhibition.

Human CatD is translated as a precursor proenzyme, and cleavage of an N-terminal peptide produces an inactive proenzyme (ProCatD) that is targeted to acidic vesicles. ProCatD is then converted to a single-chain intermediate, before final maturation into a non-covalently associated heavy chain and light chain. Pro-, single-chain, heavy-chain and light-chain CatD migrate as ∼53, 48, 33 and 14 kDa bands by SDS–PAGE^36^, respectively, which suggests that PF-7802 primarily labelled the CatD heavy chain. To confirm the identity of the 30 kDa band, we knocked down CatD using siRNA methods and found that PF-7802 labelling of this band was significantly reduced in si-CatD cells (Fig. 2b and Supplementary Fig. 4). We also analysed a CatD western blot of PF-7802-labelled ARPE-19 cells that were lysed, conjugated to biotin-TAMRA-azide, and enriched with streptavidin (Supplementary Fig. 4). This analysis showed selective enrichment of only the CatD heavy chain. In addition, we performed an *in vitro* photoaffinity labelling experiment with purified mature CatD and proCatD (Supplementary Fig. 5). We found that purified mature CatD was specifically labelled by PF-7802 and this labelling was competed by pretreatment with PF-9283 in a concentration-dependent manner. In contrast, PF-7802 labelling of proCatD was weaker, and could not be competed by pretreatment with PF-9283, suggesting non-specific labelling. Collectively, this evidence suggests that mature two-chain CatD, the most abundant and active form of CatD in cells^37^, was the primary specific target of PF-7802.

### Identification of PF-7802 site of labelling in CatD

The selective labelling of CatD by PF-7802 was not anticipated given its profile in substrate-based assays with purified proteins, in which CatD inhibition was considerably weaker than inhibition of BACE1 and BACE2 (IC_50_ values of 1.2 μM, 44 nM and 120 nM, respectively). A potential explanation for the selective labelling is that CatD is more highly expressed in ARPE-19 cells than are other aspartyl proteases. However, PF-7802 labelling in HEK293T cells overexpressing BACE1 was indistinguishable from a mock transfected control, while robust labelling of CatD was observed in cells overexpressing this enzyme (Fig. 2c). As neither binding nor abundance completely explains the lack of observed BACE1 photolabelling, we hypothesized that differences between the BACE1 and CatD active sites might change the efficiency of diradical insertion to further favour labelling of CatD. To investigate this possibility, we analysed chymotrypsin-digested PF-7802-labelled purified CatD by mass spectrometry (Supplementary Fig. 6). We matched peptides for 97% of the CatD heavy chain, only one of which was covalently modified by PF-7802. Analysis of this peptide further localized the labelling to ^307^MGMD^310^ (Fig. 2d and Supplementary Fig. 7).

Others have demonstrated that the diradical generated on ultraviolet irradiation of a benzophenone preferentially inserts into methionine residues, sometimes resulting in a marked increase in photolabelling efficiency when methionines are present proximal to the probe-binding site^38^,^39^. We generated mutants in which either CatD M307 or M309 was replaced by alanine (Fig. 2e) and overexpressed the mutants in HEK293T cells. PF-7802 labelling of the M307A mutant was reduced compared with wild-type CatD and the M309A mutant, implicating M307 as the site of covalent attachment. To further investigate the binding site, we modelled PF-9283 binding to BACE1 based on the co-crystal structure of BACE1 with a closely related analog of PF-9283 (PDB accession code: 4XXS)^26^ and aligned the apo crystal structure of CatD^40^ (PDB accession code: 1LYA, Supplementary Fig. 8). M307 was located on the periphery of the active site and corresponded to S325 in BACE1; no additional methionine residues were found within the active site of BACE1. The lack of methionine residues in close proximity to the corresponding benzophenone radical in BACE1 might lower the cross-linking efficiency to BACE1 relative to CatD—potentially contributing to the unexpected specificity of PF-7802.

### Development of a CatD-binding assay in live ARPE-19 cells

Relative protein expression levels and cross-linking efficiency may rationalize the unusual selectivity of PF-7802 for CatD over BACE1. However, these factors cannot account for the ability of weakly potent CatD inhibitors, such as PF-9283 and LY2811376, to compete probe labelling almost as well as CP-108671, which inhibits CatD with nanomolar potency (Supplementary Fig. 3). We hypothesized that the ubiquitous basic amines of BACE1 inhibitors, which are required to effectively engage the active site aspartate residues^9^, might also be causing compound accumulation in acidic compartments and particularly in lysosomes, where CatD is active^40^. Basic cathepsin K inhibitors are known to non-specifically accumulate in lysosomes^1^,^41^, which drastically changes their selectivity in whole cells. CatD selectivity cannot be measured accurately in whole cells because currently available cellular assays either require overexpression of CatD^42^ or cannot distinguish between CatD and other aspartyl proteases^43^, or CatD and other cathepsins^44^. We felt this technological gap needed to be addressed as inhibitors often show variable potency differences when tested against BACE1 in purified form compared with BACE1 endogenously expressed in live cells^45^; however, off-target potency, including CatD inhibition, is currently evaluated only with purified enzymes^9^,^10^.

**Figure 3:**
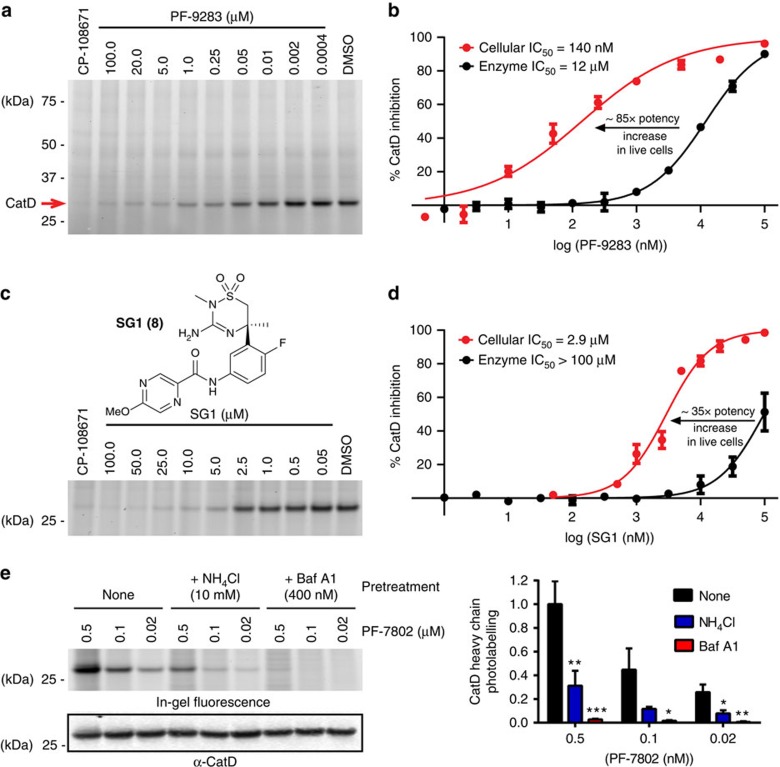
CatD cellular assay results with selected BACE1 inhibitors. (**a**,**b**) Representative in-gel fluorescence analysis (**a**) and quantification (**b**, red) of PF-9283 inhibition of cellular CatD. Cellular IC_50_ values were determined by pretreating live ARPE-19 cells with a concentration-response of PF-9283, followed by incubation with PF-7802 (100 nM, 30 min), ultraviolet irradiation, click chemistry with TAMRA-azide, and in-gel fluorescence analysis. For **b**, note potency increases of CatD IC_50_ values collected in live cells (red) compared with those collected in a substrate-based assay using purified protein (black). (**c**,**d**) Structure of sulfonyl guanidine BACE1 inhibitor (SG1, **c** top), representative gel slice from in-gel fluorescence analysis (**c**, bottom) and quantification of SG1 cellular CatD inhibition (red, **d**) compared with inhibition of the purified protein (black, **d**). (**e**) Live ARPE-19 cells pretreated with lysosome-neutralizing agent NH_4_Cl (10 mM, 2 h) or V-ATPase inhibitor bafilomycin A1 (Baf A1, 400 nM, 4 h) showed a reduction in PF-7802 labelling of CatD (top, representative in-gel fluorescence scan). PF-7802 labelling was normalized to CatD heavy chain protein level (bottom, CatD western blot); significant changes from non-pretreated samples for each PF-7802 concentration are indicated: *P<0.05, **P<0.01, ***P<0.001 using two-tailed *t*-test. For full gel of Fig. 3e, see Supplementary Fig. 12. For assay results with other BACE1 inhibitors and a summary of aspartyl protease activities of selected inhibitors see Supplementary Fig. 10 and Supplementary Table 4, respectively. For **b**, **d** and **e**, data are presented as mean±s.e.m. for three independent experiments.

We hypothesized that PF-7802, which labels CatD in live cells, could be leveraged to evaluate the cellular CatD potency of small molecule inhibitors and hence allow for cellular selectivity profiling of BACE1 inhibitors. To this end, we first performed a time course with 100 nM PF-7802 in ARPE-19 cells. A 30 min incubation with PF-7802 was selected, as it resulted in robust but incomplete labelling of CatD (Supplementary Fig. 9) and could therefore be used to evaluate reversible inhibitors^46^. We then pretreated cells with PF-9283 (400 pM–100 μM) and evaluated competitive binding with PF-7802 by in-gel fluorescence (Fig. 3a). PF-9283 concentration-dependently inhibited labelling of CatD by PF-7802, with an IC_50_ value of 140 nM (Fig. 3b). Remarkably, this value was ∼86-fold lower than that measured for PF-9283 in a substrate-based assay using purified CatD (IC_50_=12 μM). A sulfonyl guanidine BACE1 inhibitor^47^ (SG1, **8**, Fig. 3c,d), which was inactive against purified protein (IC_50_>100 μM) had quantifiable inhibition in our cellular assay (IC_50_=2.9 μM). We evaluated six additional BACE1 inhibitors including both first-11 and second-generation^34^ Eli Lilly inhibitors, BACE1 inhibitor IV (ref. 24), AMG-8718 (ref. 15), and two analogs of PF-9283 (Supplementary Fig. 10). For comparison purposes, IC_50_ values against purified aspartyl proteases and BACE1 cellular activity were determined using our in-house biochemical assays (Supplementary Table 4 and Supplementary Methods).

The measured cellular CatD IC_50_ values ranged from 59 nM for LY2811376 to 6.8 μM for LY2886721, with these two inhibitors also showing the broadest range of disconnects between cell-free enzyme and cellular assays (95-fold and >15-fold, respectively). While all tested compounds also inhibited BACE1 more potently in cells than in purified form, the shifts for CatD were larger, resulting in an effective decrease of the BACE1/CatD selectivity window (comparing cellular inhibition of BACE1 cleavage of APP to inhibition of CatD labelling by PF-7802). For PF-9283 and LY2811376, the BACE1 selectivity over CatD narrows to 23-fold for PF-9283 and to near-equivalence for LY2811376. For more CatD-sparing BACE1 inhibitors such as LY28116721 or SG1, the selectivity window narrows, but is still >2,000-fold. All four BACE1 inhibitors that have been shown to induce ocular toxicity *in vivo* (LY2811376 (ref. 11), BACE1 inhibitor IV (ref. 23), AMG-8718 (ref. 12) and PF-9283), had CatD cellular IC_50_ values of <300 nM. One potential caveat with comparing compound potencies using IC_50_ values instead of *K*_i_ values is that IC_50_s can depend on the experimental conditions. However, given that our CatD biochemical assay uses low enzyme and substrate concentrations (0.5 and 150 nM, respectively), and the compounds tested were not tight-binding CatD inhibitors (cell-free IC_50_>1 μM), it is unlikely that the results would be different using *K*_i_ values.

Potency changes appeared to be driven by the whole-cell environment, as IC_50_ values determined for these compounds by competitive PF-7802 labelling of purified CatD enzyme were within two-fold of results from a cell-free substrate-based assay (Supplementary Fig. 11). Potency increases of BACE1 inhibitors for CatD in cells roughly correlated with higher p*K*_a_, which would be expected if there was compound accumulation in acidic lysosomes (pH ∼5) (ref. 1). CatD has been found to comprise as much as 10% of the proteome in lysosomes^37^, and high local concentrations of CatD, in addition to accumulation of compounds in lysosomes, could increase compound binding to CatD. Consistent with this possibility, we found that cells treated with the neutralizing agent ammonium chloride (10 mM, 2 h) or the vacuolar H^+^-ATPase inhibitor bafilomycin A1 (400 nM, 4 h) before labelling with PF-7802, showed significantly decreased labelling of CatD without corresponding changes in CatD protein level (Fig. 3e). CatD is most active at acidic pH^48^ and the decreased CatD labelling could also result from a substantially lower binding of PF-7802 to inactive CatD under neutralizing conditions. Other active site-directed, affinity-based probes have been shown to selectively label their catalytically active targets^29^,^30^.

### BACE1 inhibitors cause accumulation of CatD protein

**Figure 4:**
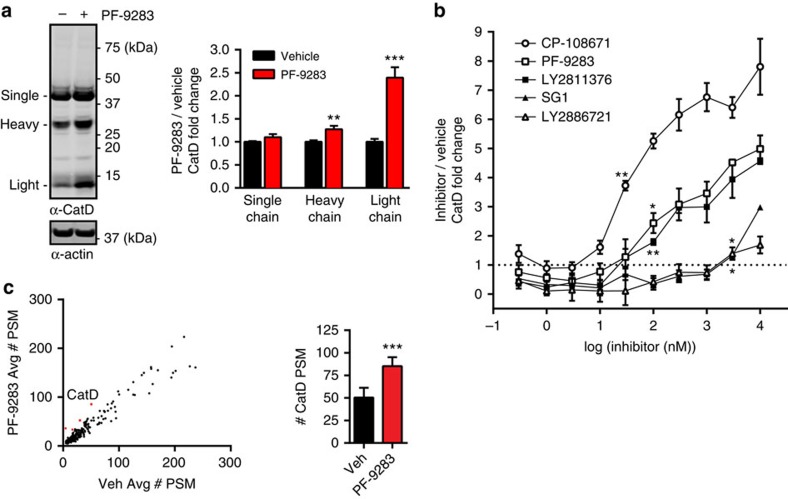
Chronic treatment with BACE1 inhibitors causes accumulation of CatD. (**a**) Representative western blot of brain homogenates prepared from mice dosed daily for 30 days with PF-9283 (80 mg kg^−1^) showed increased levels of CatD heavy and light chains, but not the CatD single chain, compared with a vehicle control. Data are presented as mean (normalized to actin)±s.e.m.; *n*=5 (PF-9283) and *n*=6 (veh) per group. (**b**) ELISA of live ARPE-19 cells incubated with BACE1 inhibitors for 7 days showed accumulation of CatD; the extent of accumulation was consistent with cellular CatD IC_50_ values (Supplementary Table 4). Data are presented as mean±s.e.m. for three independent experiments. (**c**) Proteomic analysis of RPE tissue prepared from rats dosed daily for 14 days with 80 mg kg^−1^ PF-9283. Peptide spectral matches (PSM) of 11 of 391 identified proteins were changed more than 1.5-fold (*P*<0.01, red symbols, see also Supplementary Table 5) compared with vehicle controls including a 1.7-fold increase in CatD (***P=0.0002). Average PSM were calculated from six LC-MS runs consisting of two technical replicates of *n*=3 RPE tissues per group. For **a**–**c**, significant differences were determined using a two-tailed *t*-test; *P<0.05, **P<0.01, ***P<0.001.

We next developed an additional method of measuring CatD target engagement that could also be applied *in vivo*, as a photoprobe-based assay requires ultraviolet irradiation, which cannot be performed in live animals. Probe labelling or substrate-based assays of homogenized RPE tissues from dosed animals are also problematic because homogenization and dilution would be expected to significantly reduce reversible inhibitor–enzyme interactions—particularly if the inhibitors had accumulated in acidic compartments, as we suspected may be the case for these BACE1 inhibitors. However, some studies have reported^41^,^49^ that inhibitors of cysteine cathepsins can dose-dependently increase protein levels of engaged targets, presumably due to stabilization of the bound protein to degradation. Consistent with this precedent, we found that PF-9283 significantly increased levels of CatD protein in mouse brain after 30 days of dosing (Fig. 4a), suggesting that quantitation of CatD protein increases after BACE1 inhibitor treatment might be an additional method for measuring off-target CatD inhibition.

To test this hypothesis, we incubated ARPE-19 cells with BACE1 inhibitors (300 pM–10 μM) for 7 days and then analysed CatD protein by enzyme-linked immunosorbent assay (ELISA; Fig. 4b). The levels of accumulated protein were in line with results from the cellular photoprobe assay and with previous studies of other cathepsins^41^, with CatD increases becoming significant at compound concentrations near the measured CatD cellular IC_50_ values determined using PF-7802. This effect was independent of β-secretase inhibition, as it could be reproduced with control CatD inhibitor CP-108671 (**7**) (ref. 35), which has no activity against either BACE1 or BACE2 (Supplementary Fig. 3). No significant cell death was observed at any compound concentration, and as with other cathepsin inhibitors^41^, there were no changes in CatD mRNA levels with PF-9283 treatment (Supplementary Fig. 13); this suggests that stabilization of bound protein towards proteolytic degradation could be responsible for the elevated levels of CatD. Quantitative PCR with reverse transcription also showed no mRNA changes for lysosomal enzymes cathepsin B (CTSB), cathepsin F (CTSF), glucosylceramidase (GBA) or lysosomal regulator transcription factor EB (TFEB), supporting direct engagement of CatD by PF-9283 rather than induction of general lysosomal dysfunction.

We also examined RPE tissue from rats treated with 80 mg kg^−1^ PF-9283 for 14 days (retinal histology in Fig. 1b) for increases in CatD by proteomic analysis of unenriched tryptic digests. In addition to measuring CatD levels, this method has the potential to identify other proteomic changes occurring in the RPE on PF-9283 treatment. Using peptide spectral matches (PSM) as a measure of relative abundance, we found only 11 of 391 identified proteins were changed significantly (more than 1.5-fold) in PF-9283-treated rats compared with vehicle controls (Fig. 4c, Supplementary Table 5 and Supplementary Data 2). Of those 11 proteins, only CatD has a known role in the turnover of visual proteins^16^.

### Cellular CatD IC_50_ values predict ocular toxicity

Before the first description of a BACE1 inhibitor inducing ocular toxicity^11^, CatD deficiency or deletion in mammals^17^,^18^ or in humans^19^ had been shown to cause accumulations of autofluorescent material and to induce visual impairment. In addition, it had been demonstrated that BACE1 inhibitors can also inhibit CatD, a closely related aspartyl protease^9^,^10^. However, because the BACE1 inhibitors that have been shown to induce ocular toxicity *in vivo* were advanced preclinical^12^,^23^,^26^ and clinical^11^ candidates that had already been optimized for selectivity against CatD *in vitro* (see Supplementary Table 1 and references therein), some had argued^23^ that inhibition of CatD was not likely responsible for the observed phenotype. However, our photolabelling and protein accumulation results point to CatD as a significantly underestimated off-target of BACE1 inhibitors. We wondered if the level of CatD inhibition was significant enough to account by itself for the ocular toxicity observed with certain BACE1 inhibitors. To test this hypothesis, we treated rats with the BACE1 inhibitors evaluated in our cellular assay, measured exposure of compounds and their active metabolites (Methods, Supplementary Figs 15 and 16), and evaluated H&E-stained retinal sections for ocular toxicity using epifluorescent microscopy after 7–29 days of dosing. The RPE of normal rats contained a small but variable amount of autofluorescent material typically localized along the apical border (Supplementary Fig. 17), which presumably is generated by the normal recycling of POS. In contrast, animals we considered positive for ocular toxicity exhibited an increased quantity of autofluorescent material occupying the entire cytoplasmic space from the apical to basal borders of the RPE. Generally, we did not observe dose-dependent increases in autofluorescent material, but we did find that higher compound doses resulted in an increased incidence of positive animals within a tested cohort (Supplementary Table 7), and quantitating this incidence could provide a measure of the overall severity of the ocular toxicity for each tested inhibitor.

To determine whether CatD inhibition was related to ocular toxicity, we used exposure-response analysis, which is routinely employed to link target engagement to efficacy endpoints^50^. CatD target occupancy for each study was estimated using average unbound plasma compound concentration (*C*_p,u_) and either CatD enzyme or cellular IC_50_ values (Methods). CatD target occupancy was plotted against potency (normalized for exposure) and exploratory toxicology outcomes were labelled as either ‘positive' or ‘negative' for ocular toxicity. Samples were considered positive if autofluorescent material occupied the entire cytoplasmic space from the apical to basal border in one or more regions of the RPE. Anything less than this amount was considered negative, as it falls within the range of variability that can be seen in control animals.

**Figure 5:**
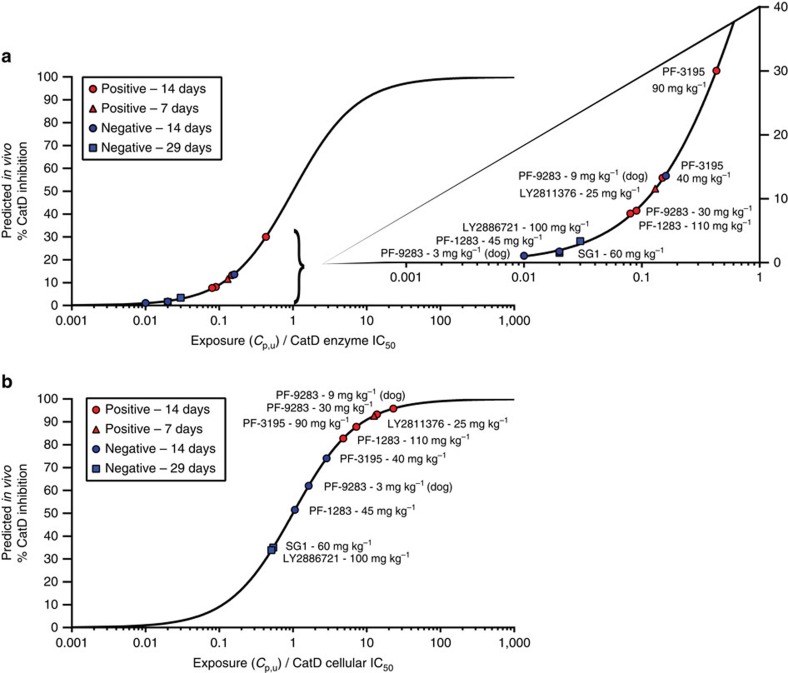
Correlations between CatD IC_50_ values and ocular toxicity *in vivo*. (**a**,**b**) Exposure-response plots comparing predicted *in vivo* CatD inhibition and ocular toxicity. CatD target occupancy was used as a surrogate to predict *in vivo* CatD inhibition and was calculated using an *E*_max_ equation based on *in vivo* average unbound plasma concentrations (*C*_p,u_) and either CatD enzyme (**a**) or cellular (**b**) IC_50_ values of selected BACE1 inhibitors (see Methods). Maximum achievable target occupancy was assumed to be 100% and where applicable, competitive binding was assumed between parent and metabolite molecules. Safety studies were conducted with daily dosing in rats with the exception of two doses of PF-9283 in dogs (indicated in parentheses). Outcomes were considered positive (red symbols) if at least one animal per cohort (*n*=4–6 for rats, *n*=2 for dogs) had accumulated autofluorescent granules in the RPE cytoplasm (as determined by fluorescence microscopy) and negative (blue symbols) if there was no ocular pathology observed. See Supplementary Fig. 17 for selected retinal histology images and Supplementary Table 7 for exposure data and incidence of ocular toxicity in each cohort.

When target occupancy was calculated using enzyme IC_50_ values (Fig. 5a), there was no apparent relationship between CatD inhibition and ocular toxicity. However, when cellular IC_50_ values were used to calculate CatD occupancy (Fig. 5b), we observed a clear separation of positive and negative outcomes in the exposure-response plot, indicating a robust relationship between target occupancy and phenotype. Notably, neither BACE1 nor BACE2 inhibition showed any correlation with ocular toxicity (Supplementary Fig. 18). In fact, the highest BACE1 or BACE2 target engagement was predicted for SG1 and LY2886721, both of which were negative for ocular toxicity, and the lowest target engagement was predicted for LY2811376, which was among the most potent inducers of ocular toxicity.

We extended our analysis to include an exploratory toxicology study in dogs dosed with PF-9283 (Fig. 5a,b—two doses, indicated in parentheses, and Supplementary Fig. 17), which also showed a relationship between ocular toxicity and the measured cellular CatD IC_50_ value. This suggests that CatD potency values generated from human ARPE-19 cells can predict CatD-driven ocular toxicity in multiple species and are not unique to specific compound metabolism or aspartyl protease biology in rat. Overall, our results suggest that effecting >80% CatD inhibition for 14 days leads to ocular toxicity. While not all compounds induced AFG accumulation at lower doses, it is possible that this would have occurred with a longer duration of treatment. However, even 29-day studies with high doses of the CatD-sparing compounds SG1 or LY2886721 generated no ocular findings, suggesting that this toxicity may be avoided with sufficiently CatD-selective BACE1 inhibitors as measured in whole-cell assays.

## Discussion

To assign a mechanism of action for a given drug, it is helpful to start with a thorough pharmacological inventory of the compound. *In* vitro activity profiling is typically limited to validated targets or homologous proteins that can be assayed in purified form. This analysis is biased to known information and cannot account for several factors that can affect drug action such as compound distribution, target processing and interacting proteins. Clickable probe analogs of drugs can agnostically identify binding proteins directly in live cells and therefore provide a valuable complement to *in vitro* substrate assays. As an added advantage, a single probe can be used for target identification and subsequently leveraged to measure target engagement of unmodified compounds, thus creating quantitative methods for assaying the newly identified targets in live cells. Here we have combined chemoproteomics with *in vivo* toxicology studies and exposure-response analysis to annotate CatD as a significant off-target of BACE1 inhibitors that cause ocular toxicity, including some that were previously thought^11^,^12^,^24^,^26^ to be selective against this enzyme.

Central to this strategy was clickable photoaffinity probe PF-7802, which allowed us to label, enrich and identify binding partners in live RPE cells. CatD was the only significant off-target detected by PF-7802, and in aggregate our data support that it is a genuine off-target of BACE1 inhibitors exhibiting ocular toxicity. PF-7802 labelling of CatD was competed by pretreatment with non CatD-selective BACE1 inhibitors^11^,^26^ or a control CatD inhibitor^35^, but not by a selective β-secretase inhibitor^34^—supporting direct target engagement of CatD. PF-7802 was found to primarily label CatD within the active site of the catalytically active form of the enzyme (30 kDa heavy chain)—suggesting that probe labelling represents specific binding to active enzyme. Further, we leveraged our photolabelling of CatD to create a selective cellular assay for this enzyme, which indicated that assays with purified protein significantly underestimate cellular potency for endogenous CatD—explaining why some of these inhibitors were previously described^11^,^12^,^24^,^26^ as exhibiting selectivity against CatD. We confirmed this unexpectedly high degree of CatD inhibition in live cells and *in vivo* using an independent measure of target engagement by quantitating CatD protein accumulation—a precedented method for detecting inhibition of other cathepsins^41^,^49^—in response to inhibitor treatment.

To assess the relationship of individual enzyme activities to ocular toxicity, we conducted a detailed series of *in vivo* exploratory toxicology studies in which cohorts of BACE1 inhibitor-treated animals were evaluated for compound exposure and accumulated RPE autofluorescent material. Exposure-response analysis indicated that a cellular assay of endogenously expressed CatD, but not a cell-free assay of the purified enzyme, was predictive of ocular toxicity. Our results suggest that BACE1 inhibitors that induce AFG increases have >80% *in vivo* target engagement of CatD. On the basis of the precedent in transgenic mice heterozygous for catalytically inactive CatD^51^, 80% inhibition of CatD alone would be more than sufficient to cause accumulation of autofluorescent material in the RPE. This level of CatD inhibition would likely have severe long-term consequences as sheep^18^ and humans^19^ with CatD loss-of-function mutations have not just visual issues, but also accumulated neuronal autofluorescent material and other symptoms of neuronal ceroid lipofuscinosis including loss of speech, impaired motor functions, and other neurodegenerative symptoms. As AD patients might need to undergo treatment with a BACE1 inhibitor for considerable periods of time, a thorough understanding and minimization of off-target CatD activity would be advisable.

In terms of evaluating a potential association between ocular toxicity and on-target engagement, we found two examples of potent BACE1 inhibitors, SG1 (ref. 47) and LY2886721 (ref. 34), that maintained >2,000-fold selectivity against CatD in live cells and displayed no ocular toxicity even after 29 days of high daily dose treatment in rats. Both SG1 and LY2886721 have very large *in vitro* selectivity windows, and therefore maintain enough selectivity in cells despite more potently inhibiting cellular CatD. Importantly, these were the most potent inhibitors of BACE1 or BACE2 among all the compounds we tested, making it unlikely that assay limitations failed to predict contributions of these BACE enzymes to POS degradation as was the case for CatD. This insight, combined with the normal retinal histology of BACE1 knockout animals that we report here and others have described elsewhere^11^,^12^, strongly suggests that inhibition of BACE1 and BACE2 does not mediate ocular toxicity. This is supported by the lack of reported ocular pathology in phase II clinical trials for LY2886721, which inhibits both BACE1 and BACE2 (ref. 34).

While our collective evidence suggests that BACE1 and BACE2 do not mediate ocular toxicity, we cannot rule out that other off-targets, in addition to CatD, could contribute to this phenotype. Unfortunately, the suboptimal physicochemical properties, poor microsomal stability and predicted poor exposure of PF-7802 make it unsuitable for *in vivo* testing. Therefore, we cannot confirm that PF-7802 causes ocular toxicity like PF-9283. In addition, while false positives of clickable photoaffinity probes can be eliminated by using carefully controlled competition experiments, potential false negatives (that is, proteins that are not labelled by PF-7802, but bind to PF-9283) are more difficult to address. We are currently working to build a suite of BACE1 inhibitor probes that will allow us to further annotate the binding partners of these compounds. This is not to detract from the value of PF-7802, which can be used to assay CatD-mediated ocular toxicity, or other (patho)physiological functions of CatD^48^ in cells. As our model predicts that off-target inhibition of CatD alone is enough to drive ocular toxicity for some BACE1 inhibitors, PF-7802 could be used to prioritize BACE1 inhibitors for advancement to *in vivo* safety studies, potentially avoiding devoting valuable resources to compounds that will likely be discontinued.

All of the inhibitors we tested displayed reduced selectivity against CatD in intact cells, potentially because compound accumulation is more pronounced in lysosomes (pH ∼5), where CatD is primarily active^48^, than in endosomes (pH ∼6), where BACE1 is believed to cleave APP^7^. CatD is also highly abundant in cells, particularly in lysosomes^37^, and high local concentrations of CatD could also contribute to reduced cellular selectivity. Further work is needed to determine whether reducing lysosomotropic properties, such as high pKa and lipophilicity^52^, increases cellular selectivity against CatD. Compound accumulation in acidic compartments has been suggested to both increase^52^ and decrease^1^,^41^ the therapeutic index of other drugs, and is one of the several possible mechanisms for disconnects between cellular and cell-free pharmacological activity. Beyond concerns about drug safety, an accurate assessment of selectivity in an appropriate context is necessary in order for small molecules to be employed as probes to interrogate biological function^53^. Our results suggest that compounds such as SG1 and LY2886721, would be preferred probes of BACE activity compared with some that have been used previously. That a poor correlation between CatD inhibition *in vitro* and ocular toxicity for BACE1 inhibitors triggered the search for additional drivers of this phenotype emphasizes the importance of assessing target engagement in the most representative biological system possible. Our collective results not only achieve this objective and designate CatD as a principal driver of ocular toxicity for BACE1 inhibitors, but also more generally demonstrate the power of cellular chemical proteomics for discerning mechanisms of drug action.

## Methods

Synthesis of novel BACE1 inhibitors and PF-7802 photoaffinity probe. See Supplementary Methods for details.

### Purified enzyme biochemical assays

Purified enzyme inhibition of BACE1, BACE2, CatD and CatE was determined using fluorescence polarization (FP) assays. Briefly, purified enzymes were treated with test compounds (1% DMSO, 37 °C) in the presence of synthetic peptide substrates (150 nM) in 30 μl total volume of NaOAc buffer (100 mM, pH 4.5, 0.0001% Tween-20) in a 384-well plate. After incubation, reactions were stopped and substrate captured by the addition of 1.5 μM streptavidin, and FP measured with an EnVision plate reader (PerkinElmer, excitation 485 nm, emission 530 nm). BACE1 activity was assessed with 0.15 nM enzyme affinity-purified from CHO-K1 cells overexpressing BACE1ΔTM96-His and the synthetic substrate Biotin-GLTNIKTEEISEISYEVEFR-C[Oregon Green]KK-OH, with a 3 h incubation time. BACE2 activity was assessed with 0.5 nM recombinant BACE2 (R&D Systems 4097-AS) and the same synthetic substrate used for BACE1, with a 1 h incubation time. CatD activity was assessed with 0.5 nM enzyme affinity-purified from human liver (Sigma C8696) and the synthetic substrate Biotin-KKPIEFFRLK-C[Oregon Green]K-OH, with a 1.5 h incubation time. CatE activity was assessed with 1.0 nM recombinant CatE (Creative BioMart CTSE-255H) and the same synthetic substrate used for CatD, with a 1.5 h incubation time.

### p*K*_a_ determination

All p*K*_a_ values were determined at Analiza, Inc. by multiplexed capillary electrophoresis (mCE) using 96-capillary array electrophoresis instrument to measure the effective mobility (migration time of the compound in relation to neutral marker, DMSO) with ultraviolet detection. The p*K*_a_ values were obtained from the ionization curves by plotting the compound effective mobility (24 points across pH range 1.7–11.2) versus pH. All p*K*_a_ results were determined using aqueous buffers with method average absolute error 0.12.

### General cell culture

Cell lines were purchased from ATCC (ARPE-19 ATCC CRL-2302 and HEK-293 ATCC CRL-15733) and tested for mycoplasma using MycoAlert Mycoplasma Detection Kit Lonza LT07-318. Early passage cell lines were used and the cells were inspected by light microscopy before use. All cell culture reagents were purchased from Life Technologies unless otherwise noted. All cells were grown at 37 °C with 5% CO_2_ atmosphere in cell culture media consisting of DMEM:F12 for ARPE-19 cells or DMEM for HEK-293T cells. Media was supplemented with 10% FBS, Penicillin–Streptomycin (100 × ) and GlutaMAX (100 × ). For SILAC experiments, cells were passaged in isotopically ‘light' or ‘heavy' media at least five times before use in probe labelling experiments. Both SILAC culture media contained 1:1 SILAC DMEM and SILAC F12 (Thermo Scientific) supplemented with 10% dialysed FBS (Gemini) and Penicillin–Streptomycin (100 × ). ‘Light' media was supplemented with 100 μg ml^−1^ of L-arginine-HCl, L-lysine-HCl and L-proline-HCl. ‘Heavy' media was supplemented with 100 μg ml^−1^ [^13^C_6_^15^N_4_]-L-arginine-HCl, [^13^C_6_^15^N_2_]-L-lysine-HCl and L-proline-HCl (Cambridge Isotope Labs).

### BACE1 biochemical cellular assay

Cellular inhibition of BACE1 was determined by measuring sAPPβ, the primary cleavage product of BACE-1, in H4 human neuroglioma cells overexpressing the wild-type human APP_695_. Briefly, cells were treated with test compound (18 h, 1% DMSO) and lysed; sAPPβ levels were measured by ELISA with a capture APP N-terminal antibody (Affinity BioReagents, OMA1-03132), wild-type sAPPβ-specific reporter antibody p192 (Elan) and tertiary anti-rabbit HRP (GE Healthcare). The colorimetric reaction was read by an EnVision plate reader (PerkinElmer).

### Live cell PF-7802 labelling for in-gel fluorescence analysis

ARPE-19 cells were plated at a density of 1.2 × 10^6^ cells per well in six-well dishes 12–24 h before probe labelling. Culture media was removed completely and replaced with 1 ml of the appropriate serum-free media (no supplements) per well. Cells were then pretreated with test compounds at the indicated concentrations for 30 min (0.5% DMSO, 37 °C, 5% CO_2_). Without replacing media, PF-7802 was then added (0.1% DMSO, 100 nM unless otherwise indicated), and incubation continued for 30 min (37 °C, 5% CO_2_) before ultraviolet irradiation (15 min, 365 nm, 4 °C, custom-modified Rayonet apparatus). Ultraviolet irradiation was performed without culture plate lids for efficient irradiation, and samples were slowly rotated to prevent evaporation. After ultraviolet irradiation, cells were washed twice with Dulbecco's phosphate-buffered saline (DPBS), scraped into DPBS, centrifuged and stored as pellets at −80 °C.

### In-gel fluorescence analysis of PF-7802-labelled cells

Cells were lysed with sonication on ice in 70 μl DPBS and protein concentrations were determined using a BCA assay (Pierce). Total protein concentrations were then normalized to 1 mg ml^−1^ in 50 μl total volume before addition of 5.5 μl click chemistry mix (1 : 3 : 1 : 0.5 volume of 50 mM CuSO_4_ (Sigma) in H_2_O : 1.7 mM TBTA (Sigma) in 4:1 *t*-BuOH:DMSO : 50 mM TCEP (Hampton Research) in dPBS : 10 mM TAMRA-azide (Lumiprobe) in DMSO - mixed immediately before use. Reagent solutions can be premade and stored at 4 °C except for TCEP, which should be made immediately before use). Samples were vortexed and incubated at room temperature for 1 h, quenched with 17 μl of 4 × LDS loading buffer (Life Technologies; samples were not boiled in loading buffer) and separated by SDS–PAGE (4–12% NuPage Bis-Tris, Life Technologies). Gels were fixed in 50% MeOH/7% AcOH for 15 min, then rinsed with 40% MeOH for at least 20 min before in-gel fluorescence scanning (Typhoon Trio, excitation 532 nm, emission 580 nm). All fluorescence gels are presented in greyscale.

### Knockdown of CatD and PF-7802 labelling in ARPE-19 cells

ARPE-19 cells were transduced with control or CatD shRNA Lentivirus (MOI=3, Sigma, catalogue nos SHC002V and TRCN0000003660) according to the manufacturer's protocol. After 3-day incubation, transduced cells were selected by the addition of 2 μg ml^−1^ puromycin. After 7 days selection in puromycin, cells were labelled with PF-7802 and cross-linked as described above for live cell PF-7802 treatment. Gels were not fixed for in-gel fluorescence scanning, so that proteins could be transferred to nitrocellulose and knockdown confirmed by western blot with an α-CatD antibody (R&D Systems AF-1014) and an α-actin antibody (Millipore 04-1040). Immunoblots were scanned using a LiCor Odyssey.

### Aspartyl protease constructs and site-directed mutagenesis

Myc-DDK-tagged BACE1 and CatD were purchased as complementary DNA clones in the pCMV6-Entry vector (OriGene, catalogue nos RC209115 and RC200719). Site-directed mutagenesis was performed using the QuikChange II kit (Agilent) according to the manufacturer's protocols. For CatD M307A, forward primer 5′-ggatgtccatgcccgcgaagccgctcaggc-3′ and its reverse complement primer were used. For CatD M309A, the forward primer was 5′-gtggcgggatgtccgcgcccatgaagccgc-3′. Mutagenesis was confirmed by sequencing using primers provided by OriGene with the cDNA.

### Overexpression of aspartyl proteases and PF-7802 labelling

HEK-293T cells were plated at a density of 1 × 10^6^ cells per well, allowed to adhere overnight and then transfected with Lipofectamine 3000 (Life Technologies) according to the manufacturer's protocol. After 48 h transfection, cells were labelled with PF-7802 and cross-linked as described above for live cell PF-7802 treatment. Gels were not fixed for in-gel fluorescence scanning, so that proteins could be transferred to nitrocellulose and overexpression confirmed by western blot with an α-FLAG antibody (Sigma F1804). Immunoblots were scanned using a LiCor Odyssey.

### Live cell PF-7802 labelling and CatD western blot analysis

ARPE-19 cells were plated at a density of 1.2 × 10^7^ cells in 15 cm dishes 12–24 h before probe labelling. Culture media was removed completely and replaced with 15 ml of serum-free media (no supplements) per well. Cells were then pretreated with PF-9283 (10 μM) and labelled with PF-7802 (100 nM) as described above for live cell PF-7802 treatment. Cells were lysed with sonication on ice in 500 μl DPBS, protein concentrations determined, and total protein concentrations were normalized to 1 mg ml^−1^ in 250 μl total volume. Samples were then treated with 25 μl click mix (prepared as described above for in-gel fluorescence analysis, except with biotin-TAMRA-azide instead of TAMRA-azide). After incubation at room temperature for 1 h, samples were cooled and proteins precipitated by the addition of ice-cold MeOH (4 ml), followed by cold CHCl_3_ (1 ml), vortexing, addition of cold H_2_O (3 ml), vortexing, and the phases separated by centrifugation (1,400*g*, 10 min). The top and bottom solvent layers were then removed and the protein disc washed carefully with 1:1 MeOH:CHCl_3_ (3 × 2 ml) and resuspended with sonication in MeOH (2 ml). After the addition of CHCl_3_ (0.5 ml), protein was pelleted by centrifugation, then resuspended with sonication in 300 μl of DPBS. Samples were then diluted with 100 μl of RIPA buffer and enriched over 25 μl of Streptavidin Plus UltraLink Resin (Pierce) for 1 h at room temperature. The resin was pelleted by centrifugation (1,000*g*, 1 min) and washed with 0.2% SDS/PBS (5 × 1 ml), 6 M urea (5 × 1 ml) and PBS (5 × 1 ml). Proteins were eluted by heating in 4 × LDS-loading buffer (Life Technologies, 72 °C, 10 min) with 2 mM biotin and 10 × NuPAGE Sample Reducing Agent (Life Technologies), separated by SDS–PAGE, and analysed by in-gel fluorescence scanning and western blot with α-CatD antibody (R&D Systems AF-1014) as described above for CatD knockdown.

### PF-7802 labelling of purified CatD

Purified CatD (Sigma C8696, isolated from human liver) or proCatD (R&D systems 1014-AS) was reconstituted and diluted to 250 nM in NaOAc buffer (50 mM, pH 4.5, 0.0001% Tween-20). Fifty microlitres was then aliquoted to each well in a 96-well plate, and wells were treated and analysed as described above for live-cell PF-7802 labelling, in-gel fluorescence analysis and CatD cellular assay with the following exceptions: incubations were performed under a normal atmosphere with slow rotation, and after ultraviolet labelling, samples were quenched with 50 μl of 0.1 M PBS (pH 7.4) before click chemistry with 11 μl of click mix. Samples were analysed by in-gel fluorescence scanning and western blot with an α-CatD antibody (R&D Systems AF-1014) as described above for CatD knockdown.

### CatD cellular assay

Cells were treated and analysed as described above for live cell PF-7802 labelling and in-gel fluorescence analysis. The integrated optical density of each band was measured using ImageJ, normalized to 100 μM CP-108671 or DMSO, and IC_50_ values quantitated using GraphPad Prism.

### Live cell lysosome neutralization and analysis

Cells were treated and analysed as described above for live cell PF-7802 labelling and in-gel fluorescence analysis with the following exceptions: lysosomes were neutralized before probe labelling with 10 mM NH_4_Cl for 2 h, or 400 nM bafilomycin A1 (Sigma) for 4 h, and gels were not fixed for in-gel fluorescence scanning, so that proteins could be transferred to nitrocellulose and CatD protein levels measured by western blot with an α-CatD antibody (R&D Systems AF1014). Immunoblots were scanned using a LiCor Odyssey. The integrated optical density of each in-gel fluorescence band was measured using ImageJ, and normalized to the CatD heavy chain protein level, also determined in ImageJ. For imaging experiments, cells were plated on coverslips and neutralized as described above. LysoSensor DND-189 (1 μM, Thermo Scientific) was then added for 30 min, followed by a 1 h washout in fresh culture media containing the neutralization reagents. Nuclei were stained with Hoescht 33342 for 5 min (20 μM, Thermo Fisher), and cells imaged live with a Zeiss LSM 780 Laser Scanning Confocal Microscope using a W Plan-Apochromat 63 × /1.0 M27 immersion objective. A 488 nm laser (10% laser power, detection from 490 to 552 nm) and 405 nm laser (6% laser power, detection from 410 to 468 nm) were used, respective detector gains set at 700 and 878, and 12-bit images acquired. Image processing was completed in ZEN lite 2012.

### Molecular modelling of PF-9283

The model of PF-9283 was built from the cocrystal structure of BACE1 with a structurally related thioamidine inhibitor (PDB accession code: 4XXS)^26^. The crystal structure of CatD (PDB accession code: 1LYA) was structurally aligned with the BACE1 structure using the structure alignment tool in Maestro version 9.7 (Schrödinger), as previously described^54^.

### General LC-MS/MS

All LC-MS/MS supplies, software and instrumentation were purchased from Thermo Scientific unless otherwise noted.

### Live cell PF-7802 labelling for SILAC LC-MS/MS analysis

For each SILAC experiment, one 15 cm plate of light ARPE-19 cells and one 15 cm plate of heavy ARPE-19 cells (90% confluence) were separately labelled, cross-linked, and collected as described above for in-gel fluorescence analysis, with inhibitors added to the light or heavy cells (2 μM), and DMSO to the corresponding paired control, followed by PF-7802 (100 nM) to both. Light cells were pretreated with PF-9283 in three independent biological replicates, and heavy cells were pretreated with PF-9283 in an additional biological replicate (combined *n*=4). Light cells were pretreated with LY2811376, CP-108671 and LY2886721 in two independent biological replicates. Total volume of serum-free SILAC culture media (no supplements) was 20 ml.

### Sample processing for SILAC LC-MS/MS analysis

Heavy and light cells were separately lysed with sonication on ice in 300 μl DPBS with a protease/phosphatase inhibitor cocktail (Cell Signaling), protein concentrations determined, and equal total protein quantities of paired heavy and light lysates were mixed (1 ml final total volume). Samples were then treated with 110 μl click mix (prepared as described above for in-gel fluorescence analysis; final concentration of 100 μM biotin-azide (ChemPep Inc, catalogue. no. 271605), 1 mM TCEP, 100 μM TBTA (Sigma, catalogue. no. 678937) and 2 mM CuSO_4_). After incubation at room temperature for 1 h, samples were cooled and proteins precipitated by the addition of ice-cold MeOH (2 ml), followed by cold CHCl_3_ (0.5 ml), vortexing, addition of cold DPBS (1 ml), vortexing and the phases separated by centrifugation (1,400*g*, 10 min) and the protein disc washed carefully with 1:1 MeOH:CHCl_3_ (3 × 1 ml) and resuspended with sonication in MeOH (2 ml). After the addition of CHCl_3_ (0.5 ml), protein was pelleted by centrifugation, then resuspended with sonication in 1 ml of 8 M urea. Samples were then reduced with 10 mM DTT (30 min, 37 °C), alkylated with 20 mM iodoacetamide (30 min, room temperature) and denatured by the addition of 8 M urea and SDS (1.0% final concentration). Samples were then diluted with 10 ml DPBS and enriched over 100 μl of Streptavidin Agarose (Pierce, catalogue no. 20353) for 3 h at room temperature. The resin was pelleted by centrifugation (1,000*g*, 3 min), washed with 1.0% SDS/DPBS (10 ml) and DPBS (3 × 10 ml), and on-bead digestion was performed by the addition of 2 μg trypsin (Pierce, catalogue no. 90058) in 200 μl 2 M urea and overnight shaking at 37 °C. Samples were stored at −80 °C.

### LC-MS/MS and SILAC data analysis

Samples were desalted using C18 spin columns (Pierce), evaporated almost to dryness, and reconstituted in 24 μl of an aqueous solution containing 5% acetonitrile and 0.1% formic acid; 6 μl of that mixture was loaded onto a trapping column and the trapped tryptic peptides separated over a C18 column (NC0414532, C18 nanoViper, 50 μm ID × 15 cm, 2 μm, 100 Å) using an EASY-nLC 1000 equipped with an autosampler. Chromatography buffers consisted of 0.1% formic acid in water (A) and 0.1% formic acid in acetonitrile (B). Peptides were separated with a linear gradient from 1–32% B at a flow rate of 300 nl min^−1^ over 190 min. Mass spectra were acquired using an Elite Velos mass spectrometer with a Nanospray Flex ion source. MS^2^ data-dependent acquisition was run in Top 20 mode where the MS^1^ scans had a resolution of 120,000, and scanned from 380 to 2,000 *m*/*z*; data-dependent MS^2^ scans were collected in the LTQ using a traditional high-low scheme with charge exclusion applied to unassigned and charge 1.

The LC-MS/MS files were processed using the Integrated Proteomics Platform (IP2, Integrated Proteomics Applications, Inc.), applying ProLuCID for protein identification and Census for protein quantification. The samples were searched against a non-redundant human UniProt database with carbamidomethylation as a fixed modification and oxidation of methionines as a variable modification. Searches were done for both light and heavy (heavy lysine (+8.014), and heavy arginine (+10.0082)) options and data were combined after searching. The precursor mass tolerance was set to 50 p.p.m. for the MS^1^, and the fragment mass tolerance to 1.5 Da with up to three missed cleavages allowed. Final protein lists were filtered to only include peptides with a mass tolerance of <10 p.p.m. and a false positive rate at the peptide level of <1%. Replicates of inhibitor-pretreated samples were combined, and the list of identified proteins filtered to exclude those with less than five total quantified peptides and s.d. >3 between the combined replicates. For PF-9283 pretreated samples, the list of identified proteins was further filtered to exclude proteins identified in only one replicate. Data are presented as the median SILAC ratio from the combined replicates (Supplementary Data 1).

### PF-7802 labelling of CatD for site of labelling analysis

Twenty five micrograms of CatD (Sigma C8696, isolated from human liver) was reconstituted in 250 μl of NaOAc buffer (100 mM, pH 4.5, 2.2 μM enzyme). The solution was split into two 125 μl aliquots in a 96-well plate and incubated either with PF-7802 (2 μM, 1% DMSO) or DMSO. After 30 min incubation with slow rotation at 37 °C, the plate was ultraviolet cross-linked as described above for in-gel fluorescence analysis. Samples were neutralized and denatured with 125 μl of 6 M urea in 0.1 M PBS (pH 7.4), reduced with 10 mM TCEP (30 min, 37 °C), alkylated with 12.5 mM iodoacetamide (30 min, room temperature), diluted with 500 μl of 1.5 mM CaCl_2_ and digested with 2 μg chymotrypsin (Promega) with overnight shaking at 37 °C. Samples were stored at −80 °C.

### Site of labelling analysis of PF-7802-labelled CatD

Samples were desalted using a C18 spin column, evaporated almost to dryness, and reconstituted in 20 μl of an aqueous solution containing 5% acetonitrile and 0.1% formic acid; 4 μl of that mixture loaded onto a trapping column and the trapped tryptic peptides separated over a C18 column (ES800 (EASY-Spray column), PepMap C18, 3 μm, 100 Å) using an Ultimate 3000 RSLC nanosystem equipped with an autosampler. Chromatography buffers consisted of 0.1% formic acid in water (A) and 0.1% formic acid in acetonitrile (B). Peptides were separated at a flow rate of 750 nl min^−1^ with a linear gradient from 2 to 30% B over 55 min, followed by a second gradient from 30 to 70% B over 14 min. Mass spectra were acquired using a Q Exactive hybrid quadrupole-Orbitrap mass spectrometer with an EASY-Spray ion source. MS^2^ data-dependent acquisition was run in Top 10 mode where the MS^1^ scans had a resolution of 70,000, an AGC of 5e5 and scanned from 375 to 2,200 *m*/*z*; data-dependent MS^2^ scans had a resolution of 17,500, and an AGC of 2e5 with charge exclusion applied to unassigned and charge 6 and above.

The LC-MS/MS files were processed using Proteome Discoverer 1.4.0. The samples were searched using SEQUEST^55^ against the human UniProt database with carbamidomethylation as a fixed modification, and oxidation and probe modification (+562.1738) as variable modifications on each of the 20 amino acids. The precursor mass tolerance was set to 20 p.p.m., and the fragment mass tolerance to 0.8 Da with up to three missed cleavages allowed. Peptides with a false positive rate of <5% were considered in the analysis.

### Sample processing for proteomic analysis of RPE tissues

Rat RPE tissues were disrupted in 25 μl of 8 M urea, 0.1 M ammonium bicarbonate and 4.5 mM DTT by aspirating with a pipette and vigorous vortexing. Samples were reduced by heating at 55 °C (20 min), alkylated with 10 mM iodoacetamide (20 min, room temperature), diluted to 2 M urea, and digested with 0.5 μg trypsin (Promega) by overnight shaking at 37 °C. The resulting peptides were desalted and concentrated using C18 Tips (Pierce). The samples were back-loaded onto the C18 tips to remove debris from tissue disruption. Eluates were dried in a centrifugal concentrator and redissolved in 20 μl of 0.1% formic acid.

### LC-MS/MS and proteomic analysis of RPE tissues

Seven microlitres of each peptide mixture was loaded onto a trapping column and the trapped tryptic peptides separated over a C18 column (Waters Acquity 186003546, 100 μm × 100 mm, 1.7 μm) using a nanoACQUITY UPLC system (Waters). Each sample was run twice to produce two technical replicates. Chromatography buffers consisted of 0.1% formic acid in water (A) and 0.1% formic acid in acetonitrile (B). Peptides were separated with a linear gradient from 2 to 30% B at a flow rate of 300 nl min^−1^ over 202 min. Mass spectra were acquired using a LTQ Orbitrap Elite mass spectrometer with a Thermo Scientific EASY-Spray ion source. The spray voltage was set to 2.2 kV and capillary temperature was 250 °C. Resolution for precursor spectra was set to 60,000. Precursor ions were selected for fragmentation using a data-dependent top 10 method with an isolation width of 2.0 and ions were fragmented in the high-pressure cell of the ion trap by CID. The normalized collision energy was set at 40% with an activation time of 10 ms.

The LC-MS/MS files were processed using Proteome Discoverer 1.1.0. The samples were searched using Mascot^56^ against the rat UniProt database with carbamidomethylation as a fixed modification, and methionine oxidation and protein N-terminal acetylation as variable modifications. The precursor mass tolerance was set to 10 p.p.m., and the fragment mass tolerance to 0.6 Da with one missed cleavage allowed. The LC pressure during the technical replicates for 9283-treated rat #4 (female) was high and these LC-MS runs were removed from the final data analysis. PSM were compared between samples to determine if samples were sufficiently similar for a label-free analysis, and samples that significantly deviated from linearity (*r*^2^<0.8) were removed (Supplementary Fig. 14). This resulted in the removal of both technical replicates for vehicle-treated rat #4 (female), which presumably deviated due to an excess of muscle proteins in the sample (dissection of the RPE is an exceedingly delicate procedure). All keratins, known contaminants and peptides with Mascot ion scores <20 were removed from the final data analysis. In addition, all hemoglobin chains and histones were removed, as they were found to elute with poor chromatography, which artificially increased PSM for these two protein types. The final proteomic data set contained two technical replicates from three vehicle rat RPEs and three PF-9283-treated rat RPEs. Average PSM for each protein were compared between vehicle and dosed samples and significant differences were determined using a two-tailed *t*-test. To be considered in the *t*-test, proteins had to be detected in at least four of the six replicates for each condition and had to have an average of at least five PSM in both vehicle and PF-9283-treated conditions.

### Chronic ARPE-19 inhibitor treatment and CatD ELISA

ARPE-19 cells were plated in 96-well plates at a density of 2 × 10^4^ cells per well. After 12–24 h, media was removed and replaced with media containing test compounds at the indicated concentration. After 7 days of compound incubation (no media changes or additional test compound treatments), conditioned media was collected for a cytotoxicity assay, and attached cells were washed once with DPBS and then lysed on ice with gentle pipetting followed by a 20 min extraction in 50 μl in ice-cold RIPA buffer (50 mM Tris, pH 7.4, 150 mM NaCl, 1 mM EDTA, 1% NP40, 1% Na deoxycholate, 0.1% SDS) supplemented with 1 mM PMSF (Sigma) and EDTA-free protease inhibitor cocktail tablets (Roche). Cytotoxicity was determined from conditioned media using an LDH assay (Pierce 88954) as per the manufacturer's protocol. CatD protein levels were measured by ELISA (EMD Millipore Q1A29) as per the manufacturer's protocol. For both the LDH assay and the ELISA, absorbance was read on an EnVision plate reader (PerkinElmer). Data were normalized to DMSO and plotted in GraphPad Prism.

### Preparation of brain homogenates and CatD western blot

Brains were homogenized in TBS (Cellgro) with 1 mM Pefabloc (Roche) and a protease inhibitor tablet (Roche) using 5 mm stainless steel beeds and a TissueLyser (Qiagen). Homogenates were pelleted (137,000*g*), and the resulting pellets were homogenized in TBS (1% TX-100 plus protease inhibitors) with sonication. This detergent-solubilized homogenate was further ultra-centrifuged (137,000*g*), total protein concentration adjusted and 35 μg of supernatant separated by SDS–PAGE (4–12% NuPage Bis-Tris, Life Technologies). Proteins were transferred to nitrocellulose and probed with an α-CatD antibody (R&D AF1029). The resulting immunoblots were scanned and bands quantified using the LiCor Odyssey.

### *In vivo* experiments

All experiments and procedures involving animals were conducted as per the established guidelines and protocols that were reviewed and approved by the Pfizer Institutional Animal Care and Use Committee. All animals used in toxicity studies were cared for in accordance with the Guide for the Care and Use of Laboratory Animals (Institute for Laboratory Animal Resources publication, 1996, NRC Press). Animals were housed at an Association for Assessment and Accreditation of Laboratory Animal Care International accredited facility in species-specific housing. Sample number was adequately powered for exploratory toxicology studies based on consistent internal practice. Clinically acceptable animals were selected for exploratory toxicology studies and assigned to groups using a computer-assisted randomization procedure based on body weights. For the fluorescent microscopic evaluation of the eyes, animals from the control group were initially evaluated in an unblinded fashion to provide the investigator an understanding of the baseline amount of autofluorescence typical for vehicle-treated animals in the study. A subsequent blinded evaluation of all animals in the study including controls was then performed to identify individuals that were outside the normal range established by the initial unblinded control group review.

### PF-9283 treatment in BACE1 KO mice for retinal histology

Exon-1 disrupted BACE1 knockout (KO) mice^27^ were originally obtained from Lexicon Genetics (currently Lexicon Pharmaceuticals) and bred at Taconic Biosciences (NY). Mice (4–5 months old) were dosed orally once daily with 20% w/v sulfobutyl ether β-cyclodextrin (SBECD; vehicle control) or 80 mg kg^−1^ PF-9283 in 20% SBECD for 2, 4, 7, 14 or 30 days using a dosing volume of 10 ml kg^−1^ in 20% SBECD. Mice were sacrificed in a CO_2_ chamber at 1 h post final dose and eyes removed, immersion-fixed in 3% glutaraldehyde for 24 h, then transfered to 10% neutral buffered formalin until processing.

### PF-9283 treatment in mice for CatD western blot

Wild-type littermate controls of BACE1 knockout mice (4–6 months old) were dosed orally once daily with 20% (w/v) SBECD (*n*=3 males and 2 females), or 80 mg kg^−1^ PF-9283 in 20% SBECD (*n*=5 males), for 30 days in a dosing volume of 10 ml kg^−1^. Mice were killed in a CO_2_ chamber at 1 h post final dose and the brains snap-frozen and stored at −80 °C.

### PF-9283 treatment in rats for proteomic analysis of RPE

Wistar Han rats (obtained from Vital River, China, 200–250 g, *n*=2 males and *n*=2 females per group) received daily oral dosing of test compounds or vehicle at the indicated concentrations. The doses were prepared in 20% SBECD and delivered in a volume of 10 ml kg^−1^. Rats were killed in a CO_2_ chamber at 3 h after the final dose, after which the RPE was dissected, snap-frozen and stored at −80 °C.

### BACE1 inhibitor treatment in rats

Wistar Han rats (obtained from Charles River, 125–300 g, 6–9 weeks old at study start, see Supplementary Table 7 for number of males and females) received daily oral dosing of test compounds or vehicle at the indicated concentrations for 7–29 days as indicated. The doses were prepared in a solution of 0.5% methylcellulose and 0.2% polysorbate 80, or 0.5% methylcellulose and hydroxypropyl methylcellulose acetate succinate (high fine grade) and delivered in a volume of 10 ml kg^−1^ per day. Blood samples for bioanalytical analysis were collected in K_2_EDTA tubes via jugular vessel. Plasma was isolated after centrifugation and stored at −20 °C prior to analysis. Animals were given an additional dose the day after the end of the study (days 8, 15 or 30 for 7, 14 and 29 day studies, respectively) then killed by gas anaesthesia (isoflurane) followed by exsanguination ∼3 h after this final dose. Eyes were removed, immersion-fixed in 3% glutaraldehyde for 24 h, then transferred to 10% neutral buffered formalin until processing.

### BACE1 inhibitor treatment in dog

Naive male beagle dogs (*Canis lupus familiaris*, >8 months old, *n*=1 male and *n*=1 female per group) received daily oral dosing of test compounds or vehicle for 14 days at the indicated concentrations. The doses were prepared in a solution of 0.5% methylcellulose and hydroxypropyl methylcellulose acetate succinate (high fine grade) and delivered in a volume of 5 ml kg^−1^ per day. Blood samples for bioanalytical analysis were collected in K_2_EDTA tubes via jugular vessel. Plasma was isolated after centrifugation and stored at –20 °C before analysis. Animals were killed via intravenous administration of barbiturate followed by exsanguination ∼3 h after the final dose on day 15. Eyes were removed, immersion-fixed in 3% glutaraldehyde for 24 h, then transferred to 10% neutral buffered formalin until processing.

### Retinal histology

After fixation for a minimum of 2 days, each eye was sectioned in half along the mid-sagittal plane with one half being embedded cut side down in a paraffin wax block and the other half returned to fixative. Paraffin blocks were sectioned at 4–5 μm using a microtome and the resulting sections were placed on glass slides, stained with H&E and coverslipped. The slides were evaluated by epifluorescent microscopy (Zeiss filter set 17, EX BP 485/20, BS FT 510, EM BP 515-565) using a Zeiss Axioplan 2 microscope.

### LC-MS/MS assay for exposure measurements of compounds

Plasma samples were analysed after protein precipitation by treating 20 μl of sample matrix with 200 μl of acetonitrile containing internal standard. All samples were centrifuged at 3,000 r.p.m. for 5 min. Then 100 μl of supernatant was transferred to a clean plate and evaporated to dryness under N_2_. Samples were reconstituted in 200 μl of 80:20 (v:v) acetonitrile:water. Samples were analysed on Waters Acquity UPLC connected to AB Sciex API5500 QTrap MS/MS.

PF-9823 (**4**) and PF-3195 (**9**) had unexpectedly low exposures, primarily resulting from conversion to active alcohol metabolites (Supplementary Fig. 15). CatD cellular IC_50_ values (Supplementary Fig. 16) and full biochemical data (Supplementary Table 6) were obtained for the active metabolites, and this information was combined with that of their parent molecules to get an overall effect on phenotype as described for exposure-response analysis below. To further mitigate any confounding effects of metabolites, oxazole analogue PF-1283 (**10**) was tested, which was significantly more stable to oxidative metabolism.

### Measurement of plasma unbound fractions

Plasma protein binding was determined in the Rapid Equilibrium Device (RED, Thermo Scientific). Frozen plasma was warmed to 37 °C and adjusted to pH 7.4. The plasma was spiked with compound to a final concentration of 2 μM. Aliquots of spiked plasma were added to the donor (red) side, followed by addition of buffer to the receiver (white) side. The RED device was covered with a gas-permeable membrane and agitated within a humidified CO_2_ incubator (5% CO_2_, 75% relative humidity). Plasma (15 μl) and buffer (45 μl) were sampled from each insert and were matrix-matched with appropriate blank matrix (plasma with 45 μl buffer and buffer with 15 μl of plasma). Samples were protein-precipitated with acetonitrile containing internal standard, and the resulting supernatant was analysed by LC-MS/MS.

### Exposure-response analysis

*In vivo* CatD target occupancy was used as a surrogate for *in vivo* CatD inhibition and was calculated based on *E*_max_ equation (1) assuming competitive binding between parent and metabolite.


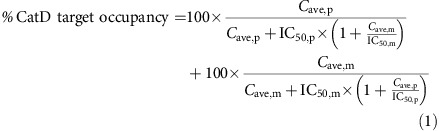


where IC_50,p_ and IC_50,m_ represent CatD potency for parent and metabolite, respectively, and *C*_ave,p_ and *C*_ave,m_ represent the average unbound plasma concentration achieved in rat exploratory toxicology studies for parent and metabolite, respectively. Maximum achievable target occupancy is assumed to be 100%. When a metabolite does not contribute significantly to CatD inhibition, *C*_ave,m_ was set to zero and the target occupancy calculation is simplified into equation (2).


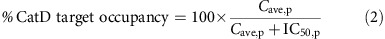


### Data availability

All relevant data are available from the authors upon reasonable request. The PDB accession code for the co-crystal structure of BACE1 with a closely related analog of PF-9283 is 4XXS, and the accession code for the apo crystal structure of human CatD is 1LYA.

## Additional information

**How to cite this article:** Zuhl, A. M. *et al*. Chemoproteomic profiling reveals that cathepsin D off-target activity drives ocular toxicity of β-secretase inhibitors. *Nat. Commun.* **7,** 13042 doi: 10.1038/ncomms13042 (2016).

## Supplementary Material

Supplementary Information Supplementary Figures 1-18, Supplementary Tables 1-7, Supplementary Methods and Supplementary References

Supplementary Data 1 Full SILAC chemoproteomics data of ARPE-19 cells enriched with PF-7802

Supplementary Data 2 Full proteomics data of RPE tissue from PF-9283-treated rats
