# Supplementary material for: Chemoproteomic profiling reveals that cathepsin D off-target activity drives ocular toxicity of β-secretase inhibitors
Source: Nat Commun. 2016 Oct 11;7:13042. doi: 10.1038/ncomms13042 (PMC5062570; doi:10.1038/ncomms13042)
Supplement: Supplementary Information — Supplementary Figures 1-18, Supplementary Tables 1-7, Supplementary Methods and Supplementary References [file ncomms13042-s1.pdf]

Supplementary Information

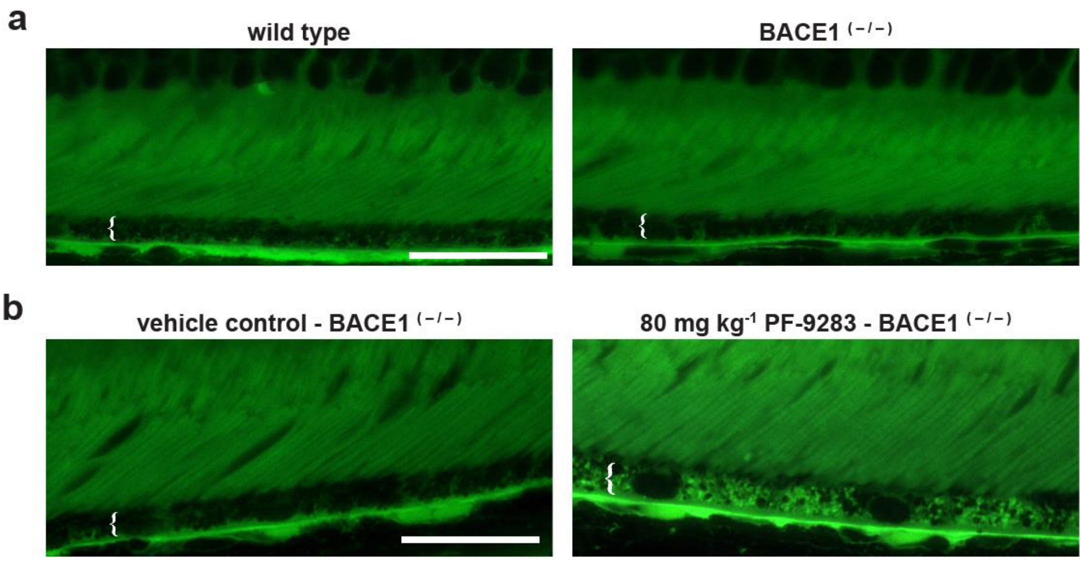

Supplementary Figure 1. Images of retinas from BACE1 $^{(-/-)}$ mice. (**a**) Representative fluorescent microscopic images of the retinas of wild type mice (left) and exon1-disrupted BACE1 knockout mice (BACE1 $^{(-/-)}$ mice, right). No changes in ocular phenotype were observed in BACE1 $^{(-/-)}$ mice. (**b**) Fluorescent microscopic images of the retinas of BACE1 $^{(-/-)}$ mice dosed daily with vehicle (left) or 80 mg kg^{-1} PF-9283 (right) for 14 days; accumulated autofluorescent granules were observed in the retinal pigmented epithelium (RPE) after PF-9283 treatment. For **a** and **b**, white bracket indicates the RPE layer. H&E stain, 40x objective, scale bar represents 50 µm, (*n* = 5 or 6 per group). For a summary of ocular findings in wild-type and BACE1$^{(-/-)}$ mice, see **Supplementary Table 2**.

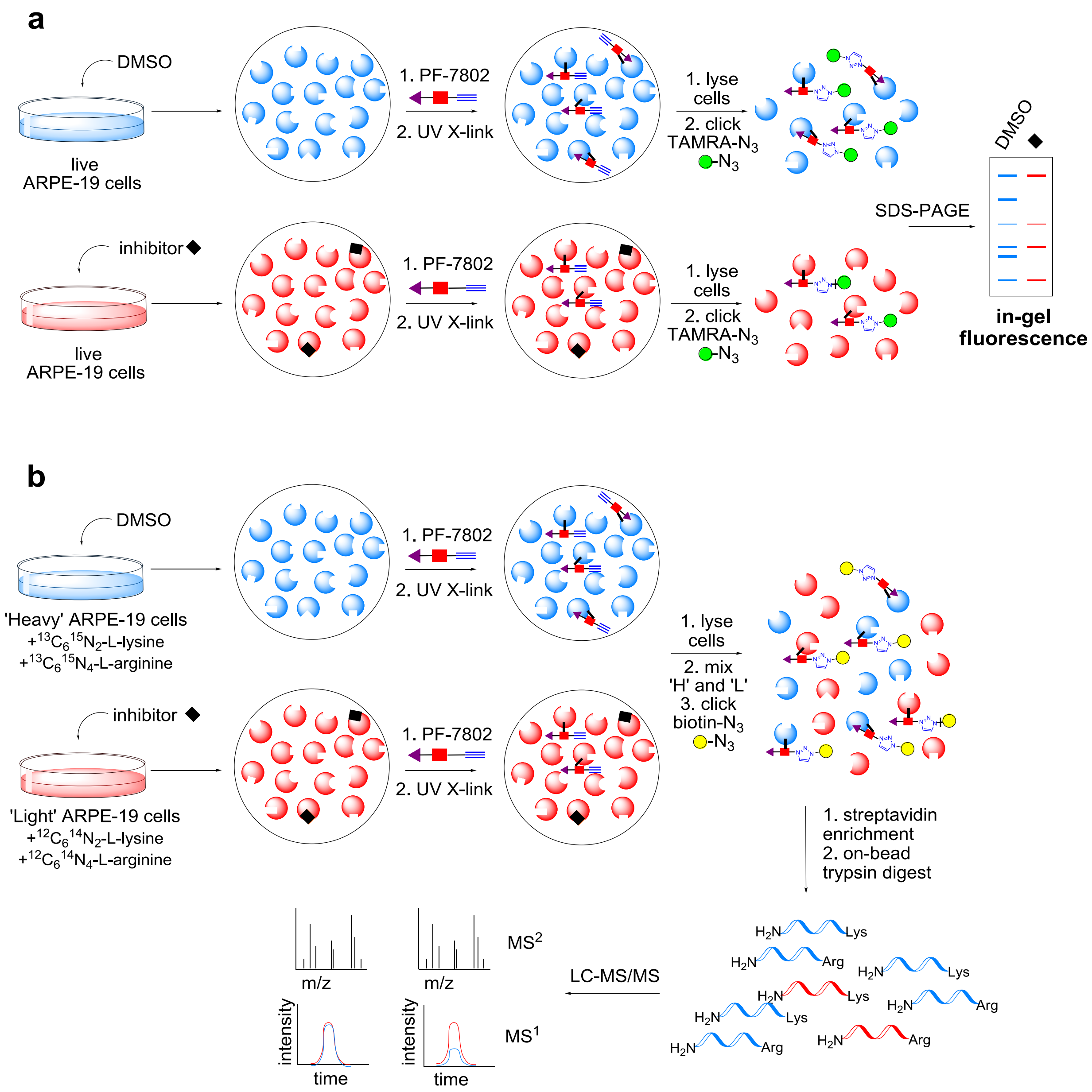

Supplementary Figure 2. Scheme for competitive chemoproteomics experiments in ARPE-19 cells. (a and **b)** Live cells are pretreated with an unmodified inhibitor (i.e., BACE inhibitor), or an equivalent volume of DMSO, before photoaffinity labeling with PF-7802. Proteins bound by the inhibitor are impeded from binding to PF-7802. For each specifically labeled protein, a decrease in signal is observed in inhibitor pretreated samples, whereas equal signal in control and inhibitor pretreated samples indicates a non-specifically labeled or enriched protein. Inhibitor binding can be detected as a decrease in fluorescence intensity after click chemistry with TAMRA-azide and analysis by SDS-PAGE (**a**, in-gel fluorescence), or a decrease in MS1 signal intensity after click chemistry with biotin-azide, enrichment of labeled proteins with streptavidin, on-bead trypsin digestion, and analysis by LC-MS/MS (**b**, SILAC mass spectrometry).

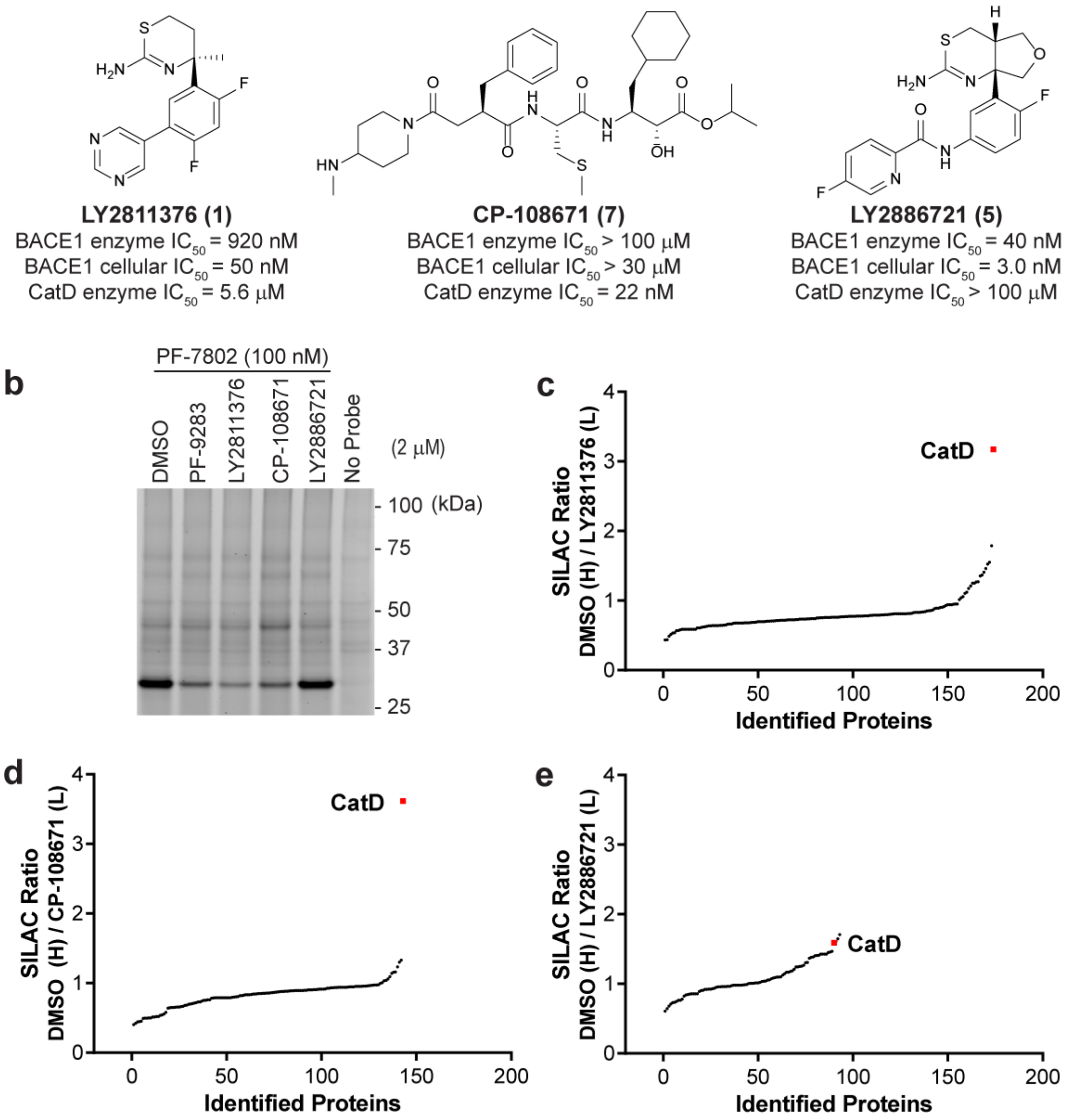

Supplementary Figure 3. **Results of chemical proteomics experiments with selected inhibitors.** (**a**) Structures of inhibitors with selected aspartyl protease activity data. (**b**) In-gel fluorescence analysis of PF-7802-labeled ARPE-19 cells. Live ARPE-19 cells were pretreated with 2 μM of the indicated inhibitors, followed by incubation with PF-7802 (100 nM, 30 min), UV irradiation, and *in vitro* click chemistry with TAMRA-azide. (**c-e**) SILAC mass spectrometry ratios for proteins identified from live ARPE-19 cells pretreated with DMSO (heavy or 'H') or 2 μM of inhibitor (light or 'L', **c** – LY2811376, **d** – CP-108671, **e** – LY2886721), and photolabeled with PF-7802 (100 nM), followed by click chemistry with biotin-azide and enrichment of probe-labeled proteins on streptavidin. Ratios are the median of at least 5 peptides per protein identification from two independent experiments. For a full list of identified proteins for all SILAC experiments see **Supplementary Data 1.**

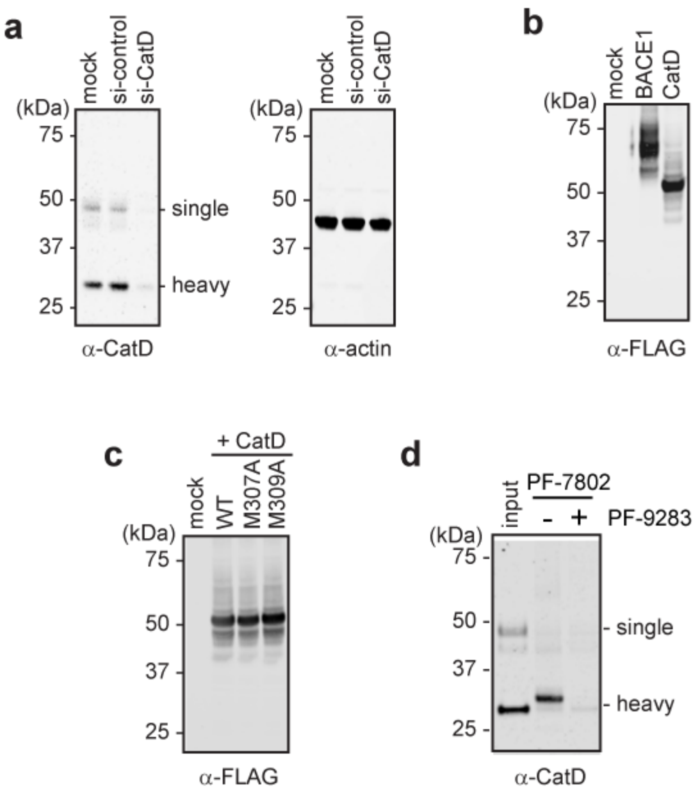

Supplementary Figure 4. **Confirmation of PF-7802 labeling of the CatD heavy chain.** (**a**) CatD western blot (left), and actin western blot (right) of **Figure 2b** samples. ARPE-19 cells were transfected with CatD siRNA (si-CatD) or control siRNA (si-control). (**b** and **c**) Full FLAG western blots of **Figures 2c** and **2d**, respectively. HEK293T cells were transiently transfected with (**b**) WT BACE1 or WT CatD or (**c**) WT CatD, CatD^{M307A}, or CatD^{M309A} (**d**) CatD western blot of enriched PF-7802-labeled ARPE-19 cells. Live ARPE-19 cells were pretreated with 10 µM of PF-9283 or an equivalent volume DMSO, followed by incubation with PF-7802 (100 nM, 30 min), UV irradiation, click chemistry with biotin-TAMRA-azide, and enrichment of probe-labeled proteins on streptavidin. For **d**, samples were compared to 12.5 µg ARPE-19 cell lysate (input).

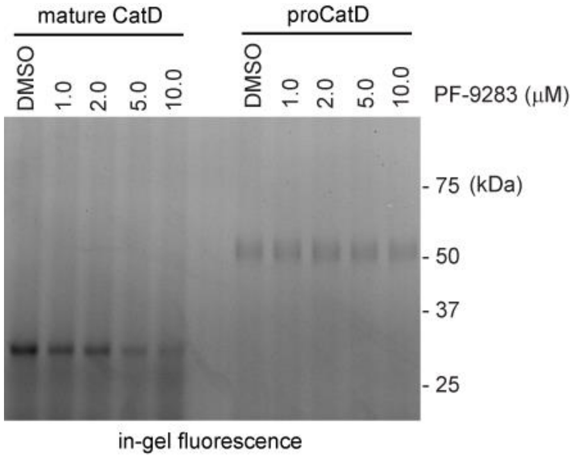

Supplementary Figure 5. **PF-7802 labeling of purified mature CatD and proCatD.** 250 nM of purified human mature CatD, or proCatD, was pretreated with a range of PF-9283 concentrations in NaOAc buffer (pH 4.5), followed by photoaffinity labeling with PF-7802 (100 nM), click chemistry with TAMRA-azide, and in-gel fluorescence analysis. PF-7802 labeling of mature CatD was concentration-dependently competed by pretreatment with PF-9283, indicating specific labeling. In contrast, PF-7802 labeling of proCatD was weaker, and could not be competed by pretreatment with PF-9283, indicating non-specific labeling.

1	[1]GPIPEVL**KN**	**YMDAQYYGEIG**	**IGTPPQCFTV**	**VFDTGSSNLW**	**VPSIHCKLLD**
51	**IACWIHHKYN**	**SDKSSTY**VKN	GTSF**DIHYGS**	**GSLSGYL**SQD	TVSVPCQ[2]SAS
101	SASA[3]L**GGVKV**	**ERQVFGEATK**	**QPGITFIAAK**	**FDGILGMAYP**	**RISVNNVLPV**
151	**FDNLMQQKLV**	**DQNIFSFYLS**	**RDPDAQPGGE**	**LMLGGTDSKY**	**YKGSLSYL**NV
201	TRKAY**WQVHL**	**DQVEVASGLT**	**LCKEGCEAIV**	**DTGTSLMVGP**	**VDEVRELQKA**
251	**IGAVPLIQGE**	**YMIPCEKVST**	**LPAITLKLGG**	**KGYKLSPEDY**	**TLKVSQAGKT**
301	**LCLSGFMGMD**	**IPPPSGPLWI**	**LGDVFIGRYY**	**TVFDRDNNRV**	**GFAEAA RL**[4]

Supplementary Figure 6. Sequence coverage of CatD from PF-7802 site of labeling analysis. Purified human CatD (2.2 µM) was incubated *in vitro* in NaOAc buffer (pH 4.5) with PF-7802 (2 µM, 30 min) before UV irradiation, digestion with chymotrypsin, and analysis by LC-MS/MS. Using Percolator, the false positive rate was set to be less than 0.05 at the peptide level. This resulted in the identification of 69 unique peptides for CatD (matched peptides shown in red) with 387 total spectral counts. Sequence coverage of the PF-7802-labeled CatD heavy chain[3-4] was 97% with MGMD identified as the only site of covalent modification (yellow). This site is not conserved in BACE1. The light chain[1-2] is also indicated, although neither gel- nor MS-based analysis identified PF-7802 labeling within this sequence. Total protein sequence coverage was 89%.

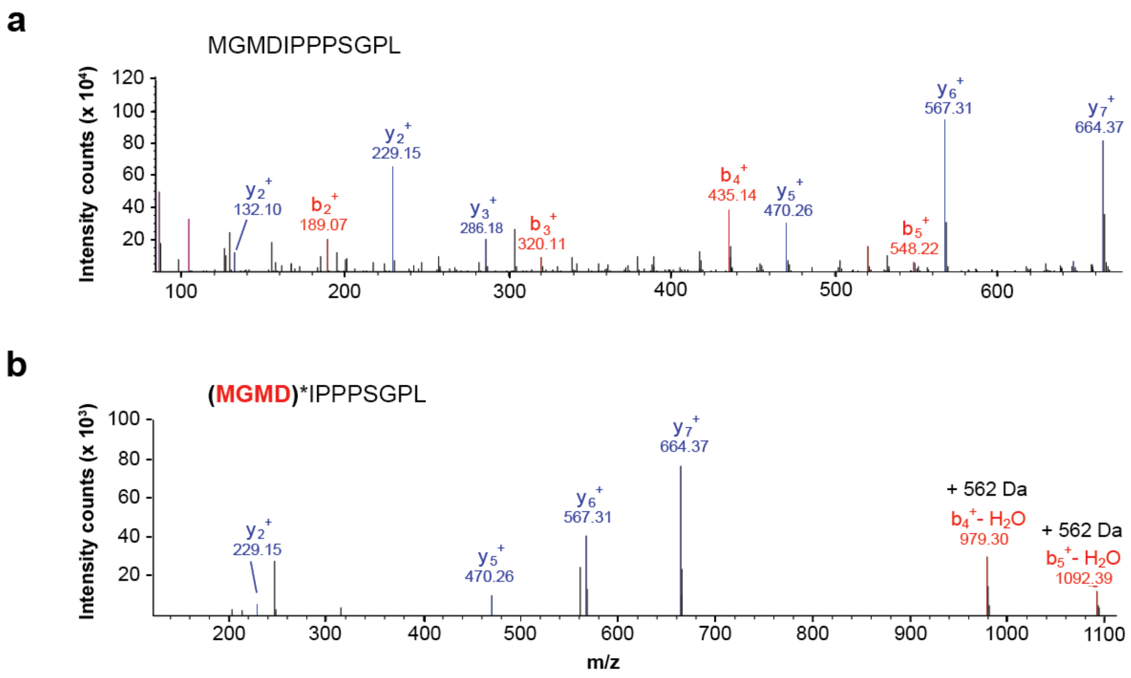

Supplementary Figure 7. Fragmentation spectrum analysis of PF-7802-labeled CatD peptide. (**a** and **b**) MS^2 fragmentation spectra of peptide MGMDIPPPSGPL without modifications (**a**) and peptide MGMDIPPPSGPL covalently modified within the sequence MGMD by PF-7802 (**b**). To generate these spectra, purified human CatD (2.2 µM) was incubated with either DMSO (**a**) or 2 µM PF-7802 (**b**) in NaOAc buffer (pH 4.5) before samples were UV irradiated, digested with chymotrypsin, and analyzed by LC-MS/MS. For **b**, + 562 Da in b_4^+ daughter ion *m/z* indicates covalent modification by PF-7802 and restricts potential labeling within the sequence MGMD.

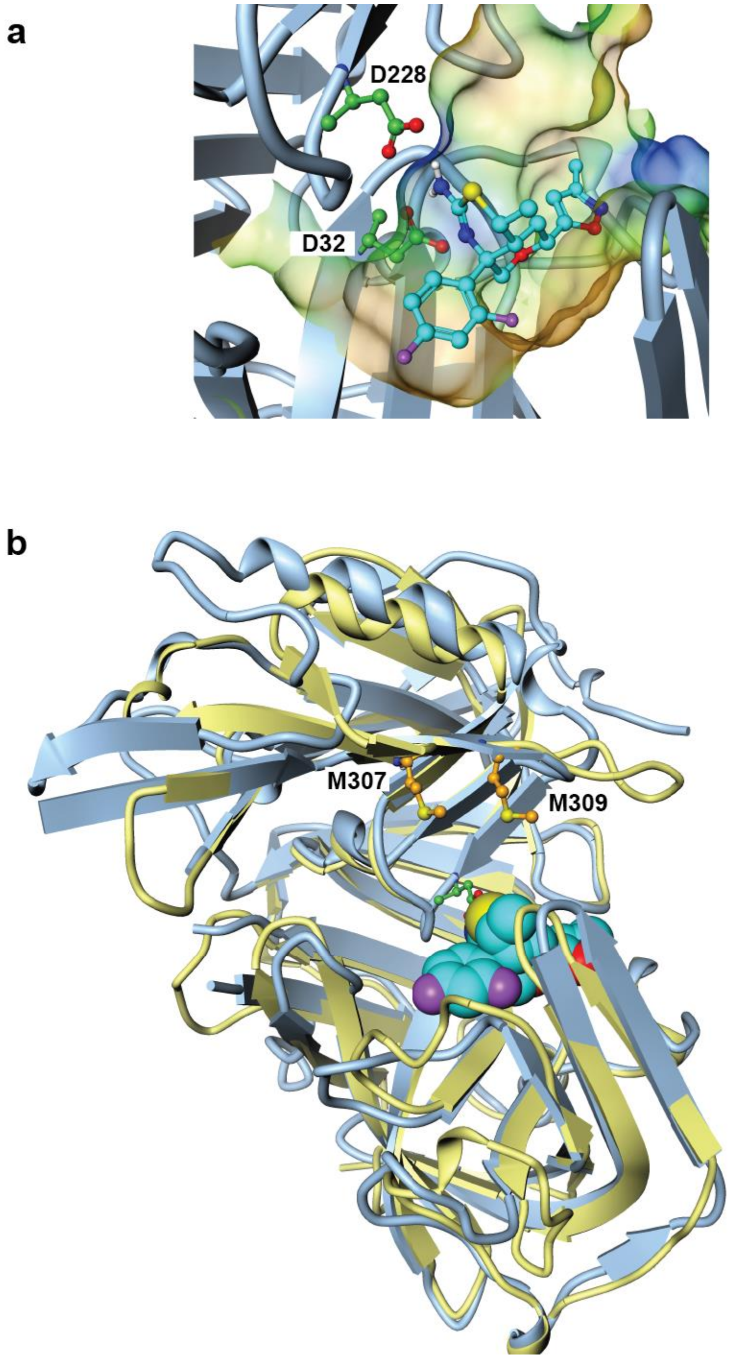

Supplementary Figure 8. Molecular modeling of PF-9283 with BACE1 and CatD. (**a**) A model of PF-9283 (cyan ball-and-stick) bound to BACE1 using PDB ID: 4XXS[1]. The catalytic acids of BACE1 (D32 and D228) are shown in green ball-and-stick. (**b**) The crystal structure of CatD (PDB ID: 1LYA, yellow) was structurally aligned with the structure of BACE1 (PDB ID: 4XXS, blue), with the PF-9283 structure shown as a CPK model. M307 and M309 of CatD are shown in orange ball-and-stick.

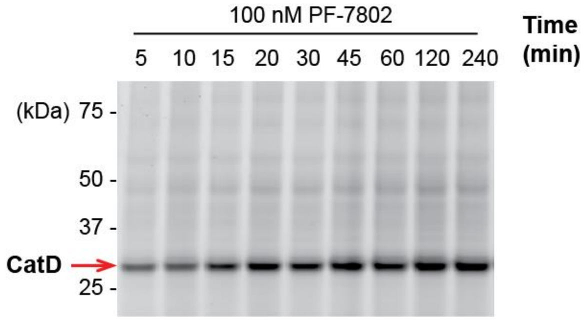

Supplementary Figure 9. Time course of PF-7802-labeling of CatD in ARPE-19 cells. In-gel fluorescence analysis of live ARPE-19 cells incubated with 100 nM PF-7802 for the indicated amounts of time before UV irradiation (15 min of irradiation, all conditions), followed by click chemistry with TAMRA-azide. At 30 minutes, the labeling of CatD was strong enough to be visualized by in-gel fluorescence analysis, but not yet saturated. These probe labeling conditions were used to determine the CatD cellular IC_{50} values found in **Figure 3** and **Supplementary Figure 10**.

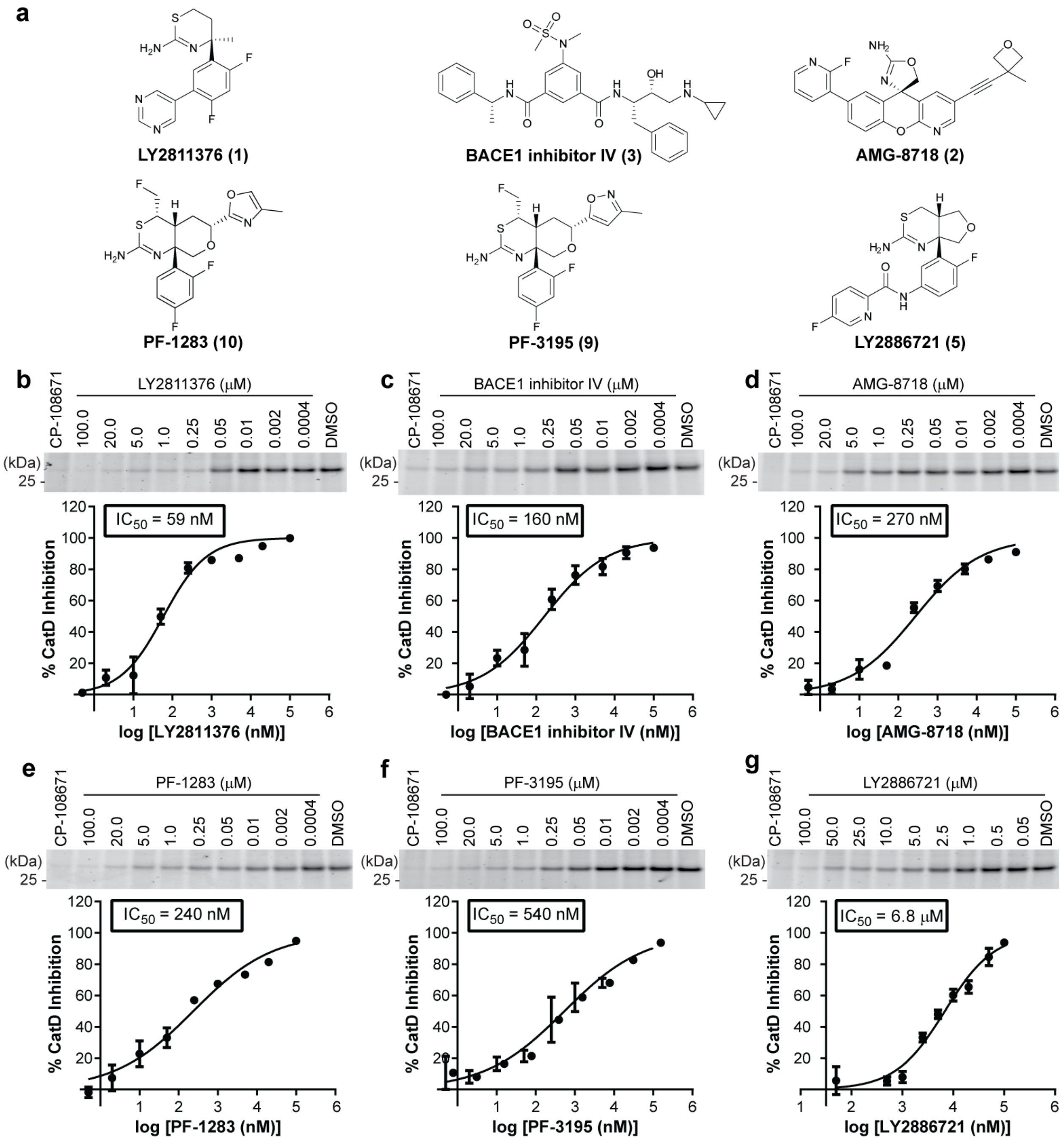

Supplementary Figure 10. **CatD cellular assay results with BACE1 inhibitors.** (**a**) Structures of BACE1 inhibitors. (**b-e**) Representative gel slice from in-gel fluorescence analysis and quantification of inhibition of cellular CatD by LY2811376 (**b**); BACE1 inhibitor IV (**c**); AMG-8718 (**d**); PF-1283 (**e**); PF-3195 (**f**); and LY2886721 (**g**). Cellular IC_{50} values were determined by pretreating live ARPE-19 cells with a range of inhibitor concentrations followed by incubation with PF-7802 (100 nM, 30 min), UV irradiation, click chemistry with TAMRA-azide, and in-gel fluorescence analysis. For a summary of aspartyl protease activities of inhibitors in **7a** above and **Figure 3** see **Supplementary Table 4**. For **b-e**, data are presented as mean ± s.e.m. for three independent experiments.

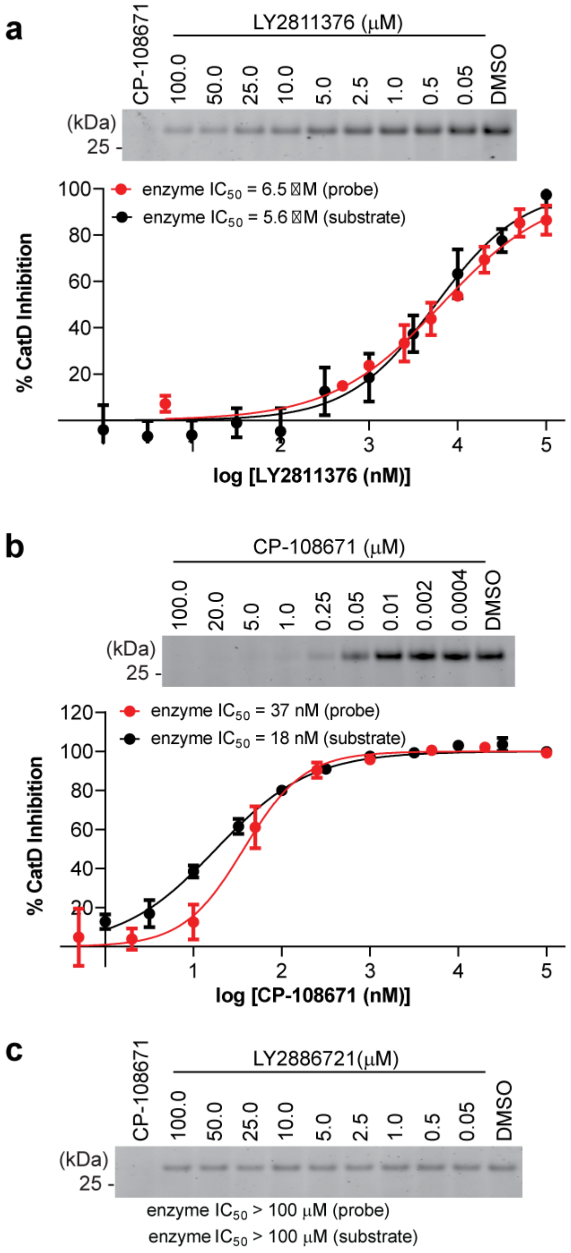

Supplementary Figure 11. Comparison of methods for determining inhibition of purified CatD. (**a**) Representative gel slice from in-gel fluorescence analysis (top) and quantification (bottom, red) of LY2811376 inhibition of purified CatD enzyme determined with the PF-7802 probe. These probe-based IC_{50} values were determined by pretreating purified human CatD (250 nM) with a range of LY2811376 concentrations followed by incubation with PF-7802 (100 nM, 30 min), UV irradiation, and click chemistry with TAMRA-azide. Note the similarity in enzyme IC_{50} values determined using the PF-7802 probe (red) compared to that determined from a biochemical substrate assay with purified protein (black), contrasting with the differences seen in **Figure 3**. (**b** and **c**) The same analysis as **a**, but with inhibitors that were potent (CP-108671, **b**) or inactive (LY2886721, **c**) in the purified CatD biochemical substrate assay. For **a** and **b**, data are presented as mean ± s.e.m. for three independent experiments.

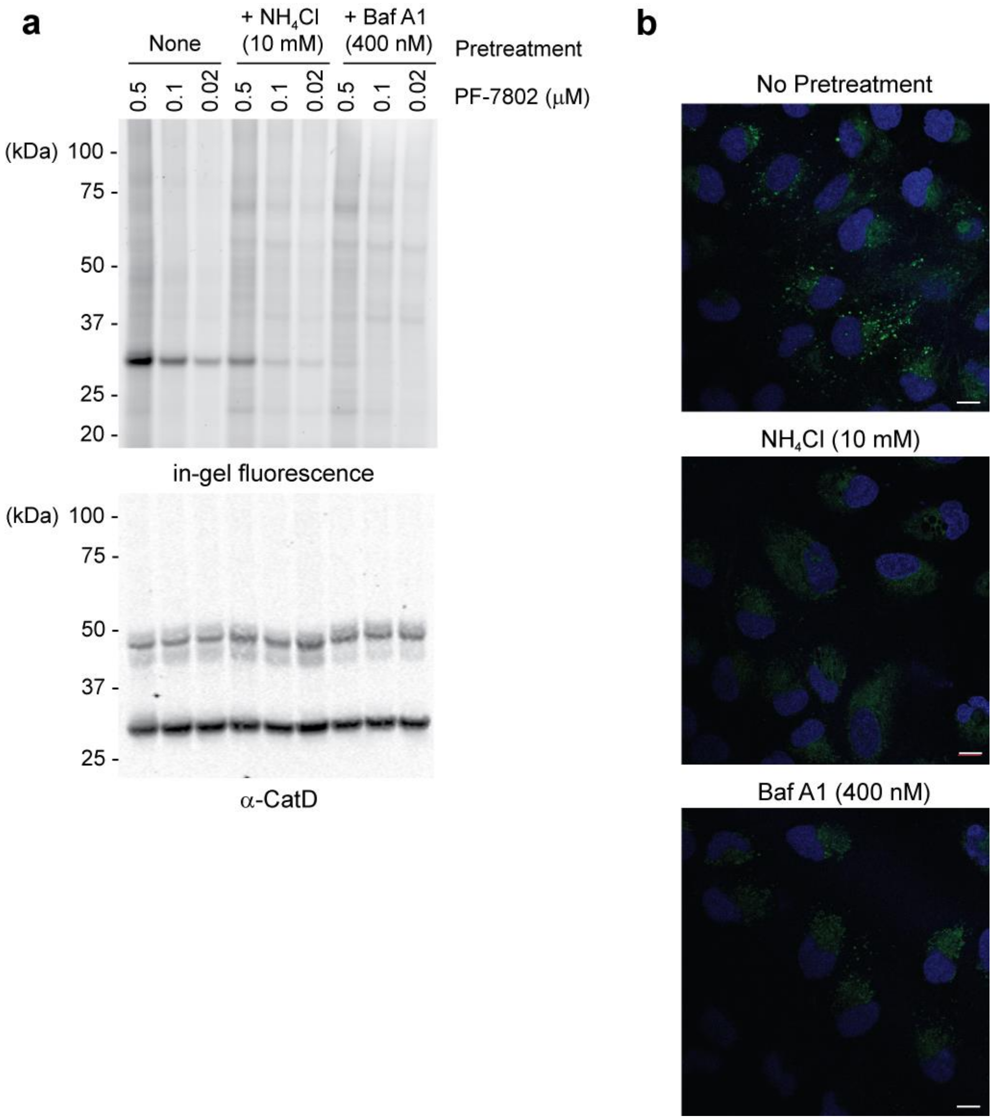

Supplementary Figure 12. **Results of lysosome neutralization experiments in ARPE-19 cells.** (**a**) Full gels of **Figure 3e**. Live ARPE-19 cells were pretreated with lysosome-neutralizing agent NH_4Cl (10 mM, 2 h) or V-ATPase inhibitor bafilomycin A1 (Baf A1, 400 nM, 4 h). Neutralized cells showed a reduction in PF-7802 labeling of CatD (**a**, top, in-gel fluorescence analysis) that was normalized to CatD heavy chain protein levels (**a**, bottom, CatD western blot). (**b**) Pretreatment with NH_4Cl and bafilomycin A1 resulted in a reduction of lysosomal pH as detected by loss of bright punctate labeling in live cell confocal microscopy with LysoSensor DND-189 (Green). Nuclei were stained with Hoescht 33342 (blue). Merged images were adjusted equally in ZEN lite 2012 software. Green channel black = 100; gamma = 1.0; and white = 2500. Blue channel black = 0; gamma = 0.45; and white = 2500. Scale bar = 10 μm.

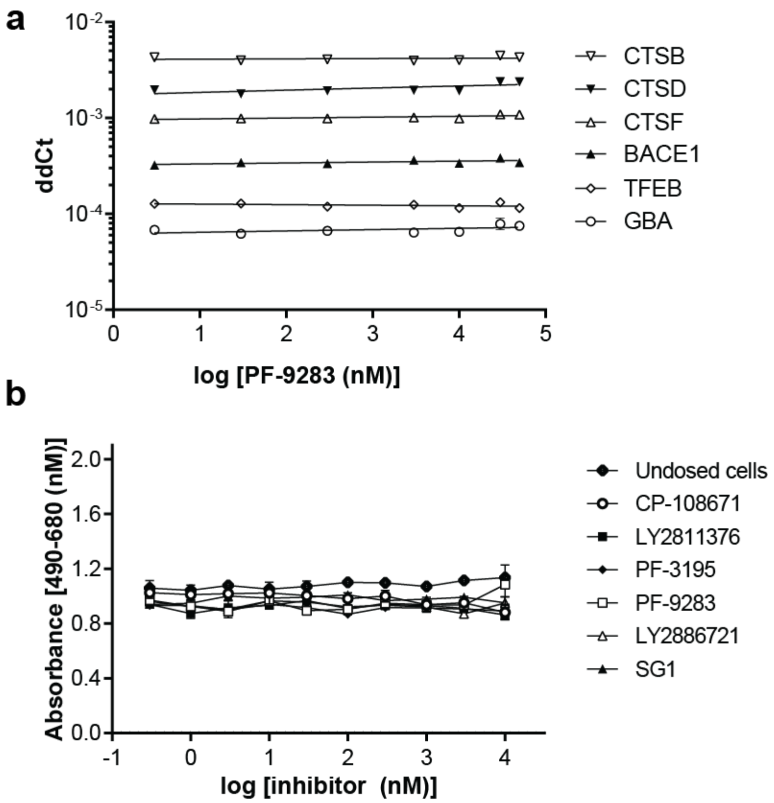

Supplementary Figure 13. qRT-PCR and cytotoxicity analysis of inhibitor-treated ARPE-19 cells. (**a**) qRT-PCR of ARPE-19 cells treated with PF-9283 for seven days did not display changes in mRNA levels of cathepsins B, D or F (encoded by genes CTSB, CTSD, and CTSF), BACE1, lysosomal regulator TFEB or lysomal enzyme glucocerebrosidase (GBA). (**b**) LDH viability assay of ARPE-19 cells treated for seven days with aspartyl protease inhibitors showed no evidence of cytotoxicity after 7 days dosing. For **a** and **b**, data are presented as mean ± s.e.m. for three independent experiments.

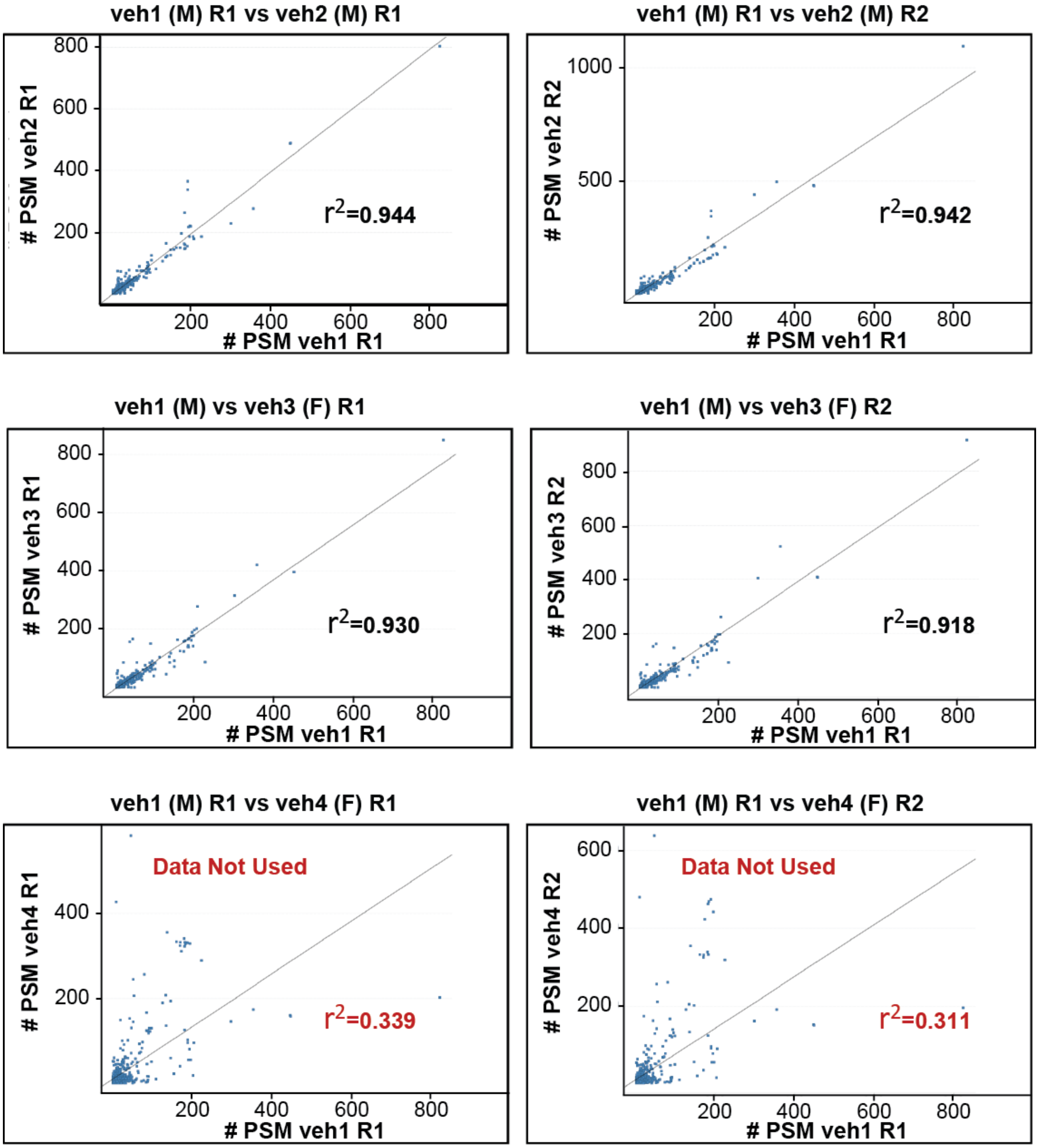

Supplementary Figure 14A. Vehicle sample comparison for proteomic analysis of RPE tissue prepared from rats. The number of spectral matches (PSM) for each protein was plotted using Spotfire (TIBCO). Vehicle sample 4 had excess muscle proteins consistent with excess tissue contaminating the RPE dissection and was removed from analysis. M = male vehicle, F = female vehicle; R1 or R2 indicates technical replicate 1 or 2. For PSM values and protein identifications, see **Supplementary Data 2**.

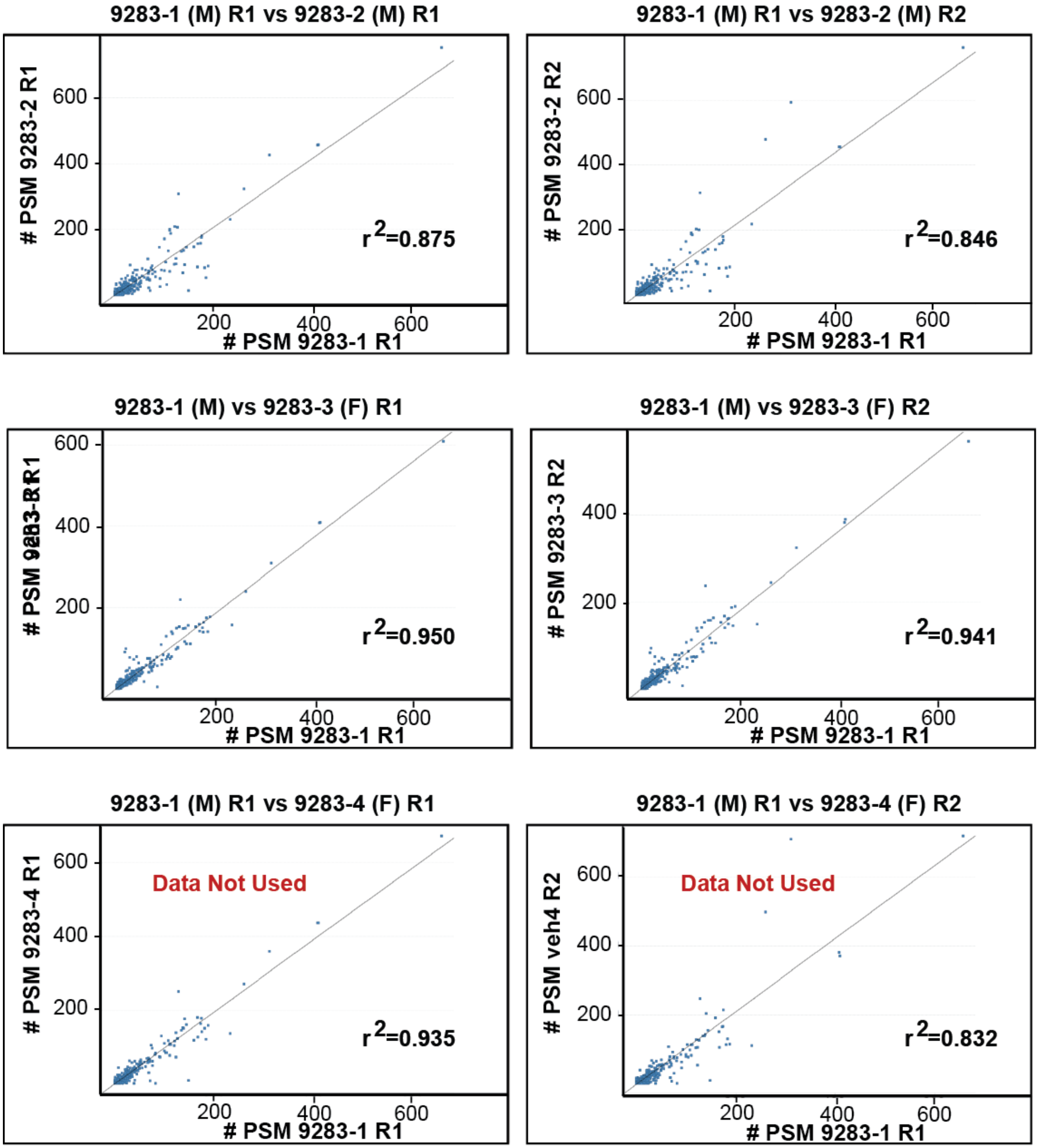

Supplementary Figure 14B. PF-9283 sample comparison for proteomic analysis of RPE tissue prepared from rats. The number of spectral matches (PSM) for each protein was plotted using Spotfire (TIBCO). 9283 sample 4 was removed from the analysis because of chromatographic difficulties including high system pressure. M = male, F = female; R1 or R2 indicates technical replicate 1 or 2. For PSM values and protein identifications, see **Supplementary Data 2**.

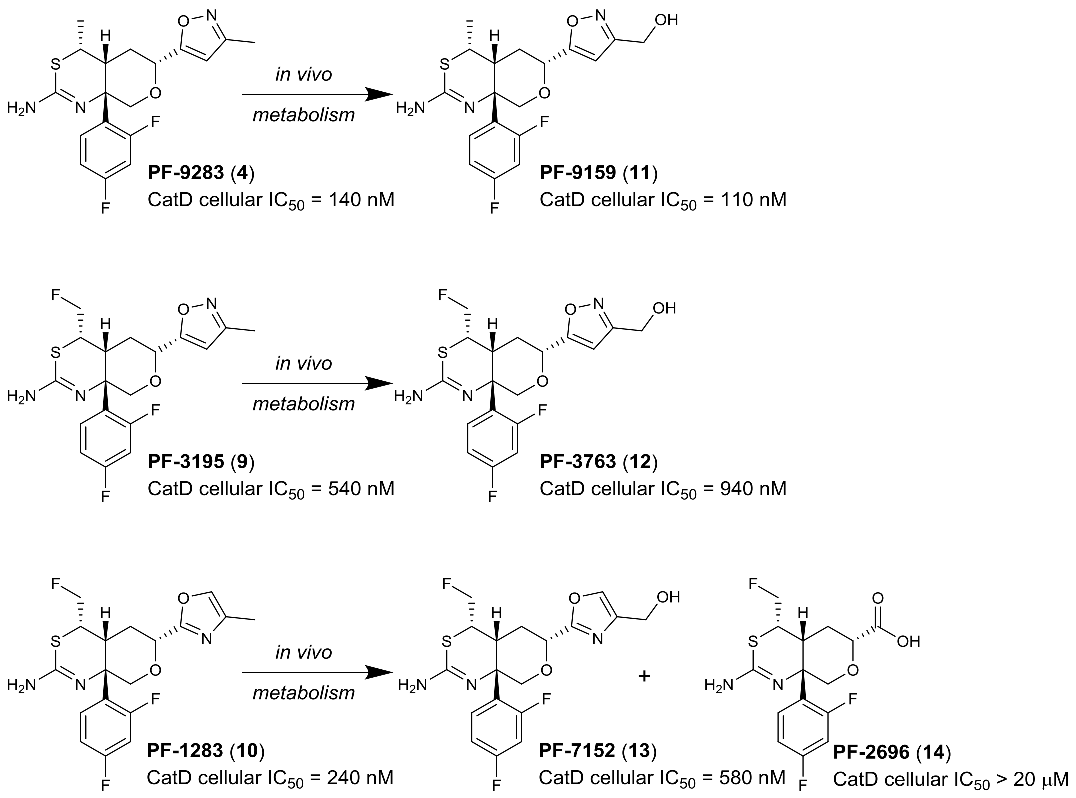

Supplementary Figure 15. *In vivo* metabolism of BACE1 inhibitors in rats. Metabolite identity was confirmed by resynthesis. Levels of both parent molecules and active metabolites were quantified during *in vivo* studies. PF-1283 was significantly less susceptible to oxidative metabolism and acid PF-2696 was the major metabolite. PF-2696 was inactive (IC_{50} > 20 µM) in BACE1, BACE2 and CatD enzyme assays as well as the CatD cellular assay. For a full comparison of inhibitor and metabolite aspartyl protease activities see **Supplementary Table 6**.

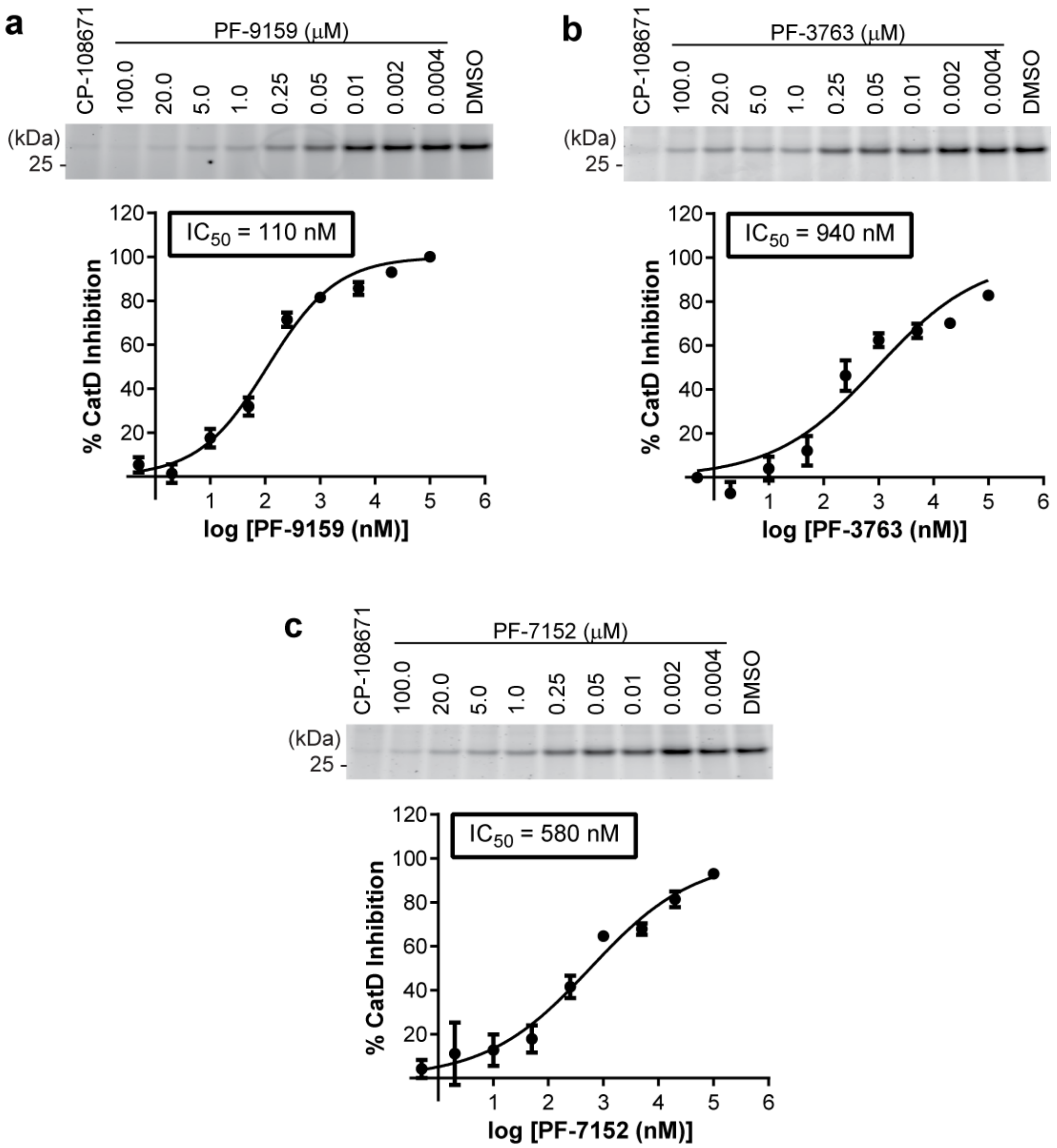

Supplementary Figure 16. **CatD cellular assay results with metabolites.** (**a**-**c**) Representative gel slice from in-gel fluorescence analysis and quantification of inhibition of cellular CatD by PF-9159 (**a**); PF-3763 (**b**); and PF-7152 (**c**). Cellular IC_{50} values were determined by pretreating live ARPE-19 cells with a range of inhibitor concentrations followed by incubation with PF-7802 (100 nM, 30 min), UV irradiation, click chemistry with TAMRA-azide, and in-gel fluorescence analysis. For a full comparison of inhibitor and metabolite aspartyl protease activities see **Supplementary Table 6**. Data are presented as mean ± s.e.m. for three independent experiments.

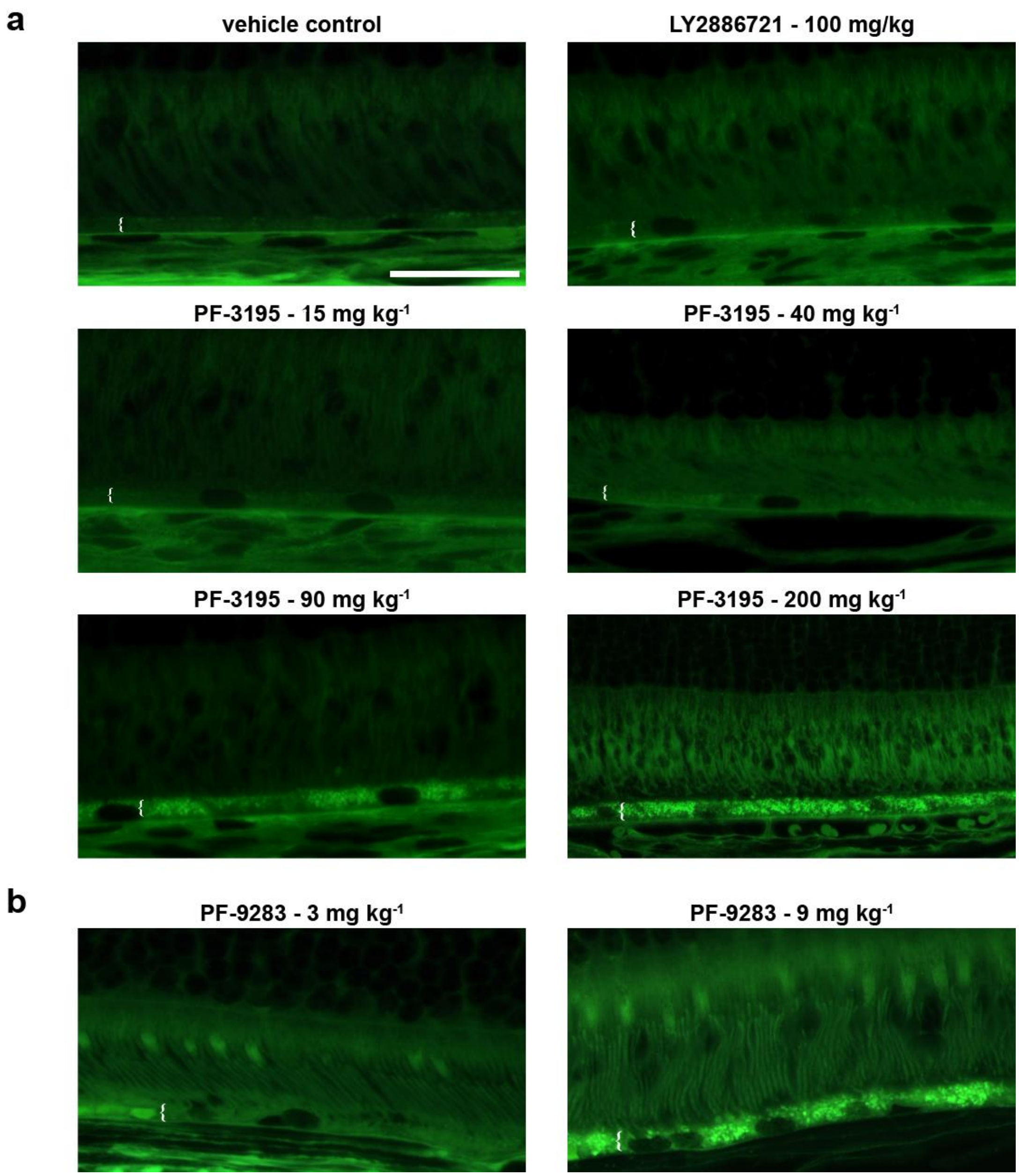

Supplementary Figure 17. Images of retinas from exploratory toxicology studies. (**a**) Representative fluorescent microscopic images of the retinas of rats dosed daily with BACE1 inhibitors. After 29 days of treatment with 100 mg kg^{-1} LY2886721, no ocular abnormality was observed. A 14 day study with daily dosing of PF-3195 showed accumulated autofluorescent granules with 90 mg kg^{-1} or 200 mg kg^{-1} dosing, but no intermediate phenotype with 15 mg kg^{-1} or 40 mg kg^{-1} dosing. (**b**) Representative fluorescent microscopic images of dogs dosed daily with PF-9283 for 14 days exhibit accumulated autofluorescent granules in the RPE after 9 mg kg^{-1} dosing, but not after 3 mg kg^{-1} dosing. For **a** and **b**, white bracket indicates the RPE layer, H&E stain, 40x objective, scale bar represents 50 µm, n = 4 - 6 for rats, n = 2 for dogs. See **Supplementary Table 7** for incidence of ocular toxicity in each cohort.

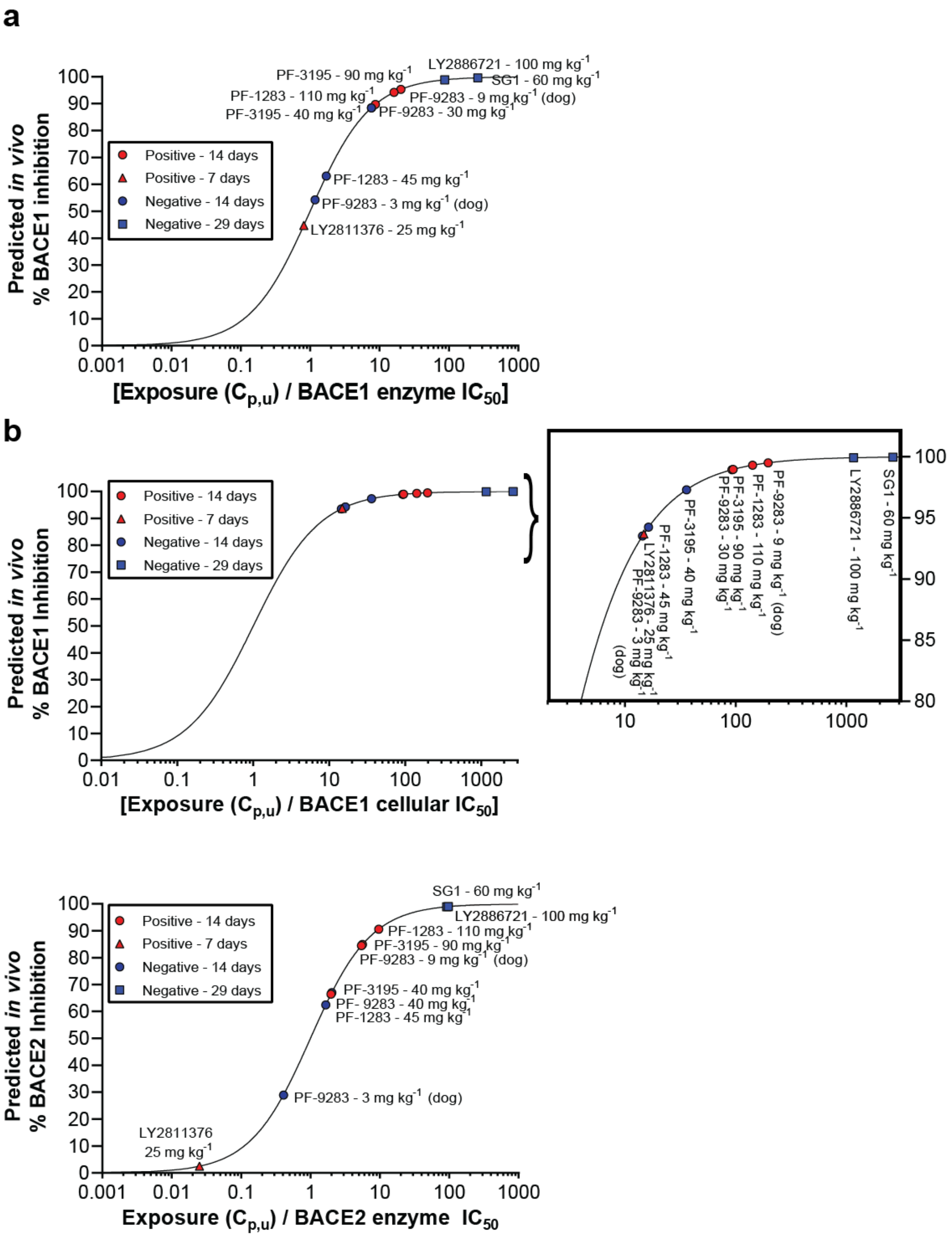

Supplementary Figure 18. Lack of correlation between BACE1/2 inhibition and ocular toxicity *in vivo*. (**a** and **b**) Exposure-response plots comparing predicted *in vivo* inhibition and ocular toxicity. Target occupancy was used as a surrogate to predict *in vivo* inhibition and was calculated using an E_{max} equation based on *in vivo* average unbound plasma concentrations ($C_{p,u}$) and either BACE1 enzyme (**a**), BACE1 cellular (**b**), or BACE2 enzyme (**c**) IC_{50} values of inhibitors. Maximum achievable target occupancy was assumed to be 100% and, where applicable, competitive binding was assumed between parent and metabolite molecules. Safety studies were conducted with daily dosing in rats with the exception of two doses in dogs (indicated in parentheses). Outcomes were considered positive (red symbols) if at least one animal per cohort (*n* = 4 - 6 for rats, *n* = 2 for dogs) had accumulated autofluorescent granules in the RPE cytoplasm (as determined by fluorescence microscopy) and negative (blue symbols) if there was no ocular pathology observed. See **Supplementary Figure 14** for selected retinal histology images and **Supplementary Table 7** for exposure data and incidence of ocular toxicity in each cohort.

Name	Structure	Reported fold selectivity for BACE1 over other aspartyl proteases					Reference
		**BACE2**	**CatD**	**CatE**	**pepsin**	**renin**	
LY2811376 **(1)**	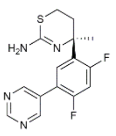	12	66	NA*	140	160	2
AMG-8718 **(2)**	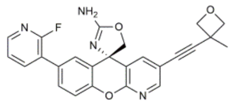	7.3	3,300	> 1,400	> 1,400	> 1,400	3
BACE1 inhibitor IV **(3)**	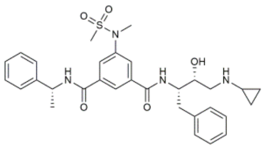	15	510	NA*	NA*	> 3,300	4
PF-9283 **(4)**	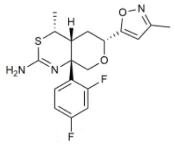	1.9**	210	320**	NA*	NA*	1
LY2886721 **(5)**	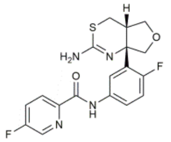	0.50	> 15,000	NA*	> 4,900	> 4,900	5

Supplementary Table 1. **Aspartyl protease selectivity of previously disclosed BACE1 inhibitors**. Selectivity was determined using purified aspartyl proteases as described in the referenced publications. * NA – not available. **Not previously disclosed.

	**WT ocular toxicity**		**BACE1$^{(-/-)}$ ocular toxicity**	
**Duration (days)**	**vehicle control**	**PF-9283 treated**	**vehicle control**	**PF-9283 treated**
2	Neg 6/6	Neg 5/5*	NA	NA
4	Neg 6/6	Pos 1/5*	NA	NA
7	Neg 6/6	Pos 6/6	NA	NA
14	Neg 6/6	Pos 5/5*	Neg 5/5*	Pos 4/5*
30	Neg 6/6	Pos 6/6	Neg 6/6	Pos 6/6

Supplementary Table 2. Summary of ocular findings in wild-type and BACE1$^{(-/-)}$ mice. Outcomes were considered positive (pos) if at least one animal had accumulated autofluorescent granules in the RPE cytoplasm as determined by fluorescence microscopy, and negative (neg) if there was no ocular pathology observed. Entries are listed as number of positive or negative animals / total number of animals evaluated (*n* = 5 or 6 per group, as indicated). PF-9283 was dosed daily at 80 mg kg^{-1}. NA: not available. * Insufficient sample available for analysis of sixth animal.

	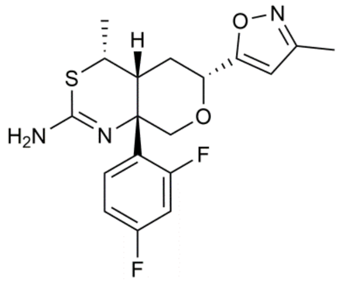 **PF-9283 (4)**	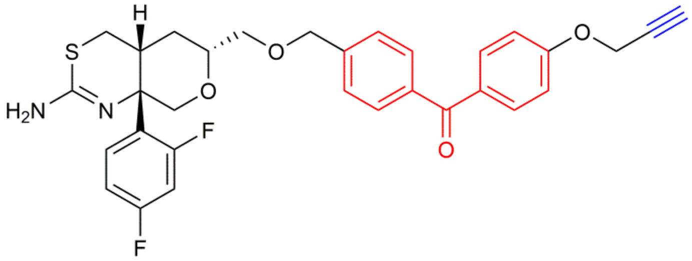 **PF-7802 (6)**
BACE1 enzyme IC_{50}	74 nM	44 nM
BACE1 cellular IC_{50}	6.0 nM	320 nM
BACE2 enzyme IC_{50}	140 nM	120 nM
CatD enzyme IC_{50}	12 µM	1.2 µM
CatE enzyme IC_{50}	24 µM	4.2 µM

Supplementary Table 3. Comparison of PF-9283 and PF-7802 aspartyl protease inhibitory activities. Enzyme IC_{50} values were determined using fluorescence polarization assays with purified human enzymes and fluorescent peptide substrates. BACE1 cellular IC_{50} values were determined by ELISA detection of sAPP-beta levels in H4 neuroglioma cells transfected with human wild-type APP. Data are presented as the mean of at least three independent experiments.

	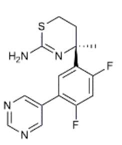	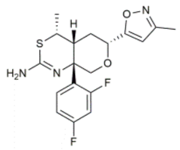	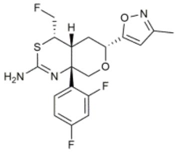	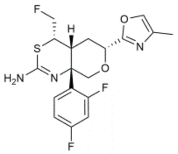	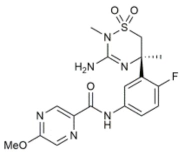	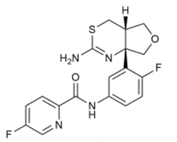
	**LY2811376 (1)**	**PF-9283 (4)**	**PF-3195 (9)**	**PF-1283 (10)**	**SG1 (8)**	**LY2886721 (5)**
CatD cellular IC_{50}	59 nM	140 nM	540 nM	240 nM	2.9 µM	6.8 µM
CatD enzyme IC_{50}	5.6 µM	12 µM	9.0 µM	13 µM	> 100 µM	> 100 µM
CatD potency increase in live cells	**95x**	**86x**	**17x**	**54x**	**> 34x**	**> 15x**
BACE1 cellular IC_{50}	50 nM	6.0 nM	15 nM	10 nM	590 pM	3.0 nM
BACE1 enzyme IC_{50}	920 nM	74 nM	62 nM	120 nM	6.0 nM	40 nM
BACE1 potency increase in live cells	**18x**	**12x**	**4.1x**	**12x**	**10x**	**13x**
BACE2 enzyme IC_{50}	29 µM	140 nM	150 nM	95 nM	17 nM	36 nM
CatD/BACE1 enzyme IC_{50}	**6.1**	**160**	**150**	**110**	**> 17,000**	**> 2,500**
CatD/BACE1 cellular IC_{50}	**1.2**	**23**	**36**	**24**	**4,900**	**2,300**
pK_a	8.9	7.7	7.0	7.0	6.9	7.6

Supplementary Table 4. Comparison of aspartyl protease inhibitory activities of selected BACE1 inhibitors. Enzyme IC_{50} values were determined using fluorescence polarization assays with purified human enzymes and fluorescent peptide substrates. BACE1 cellular IC_{50} values were determined by ELISA detection of sAPP-beta levels in H4 neuroglioma cells transfected with human wild type APP. CatD cellular IC_{50} values were determined using the PF-7802 photoprobe in ARPE-19 cells. pK_a values were determined using a capillary electrophoresis method. Data are presented as the mean of at least three independent experiments.

Description	Accession	Veh Avg. # PSM	Veh. PSM SDEV	PF-9283 Avg. # PSM	PF-9283 PSM SDEV	PF-9283/ Veh. PSM	*P* value
Isoform 3 of NADH-cytochrome b5 reductase 3	P20070-3	9.5	0.8	6.2	0.8	0.65	0.00003
Transgelin	P31232	17.3	2.3	26.3	2.8	1.52	0.0001
Cathepsin D	Q6P6T6	50.5	10.7	85.5	10.3	1.69	0.0002
Sulfated glycoprotein 1	P10960	30.0	4.6	52.5	10.7	1.75	0.0008
Serine (or cysteine) proteinase inhibitor, clade H, member 1, isoform CRA_b	Q5RJR9	29.8	6.2	17.7	1.8	0.59	0.0010
Annexin A6	P48037	14.8	3.1	9.0	2.6	0.61	0.0052
Guanine nucleotide-binding protein G(o) subunit alpha	D4ABT0	23.2	4.7	14.8	3.5	0.64	0.0059
Protein Tyrp1	D3ZH71	9.5	2.4	5.7	1.4	0.60	0.0071
4F2 cell-surface antigen heavy chain	Q794F9	16.7	6.9	6.8	2.3	0.41	0.0080
Cytochrome c oxidase subunit 6C-2	P11951	8.3	2.4	5.0	0.6	0.60	0.0086
Apolipoprotein A-I	P04639	5.0	1.4	7.8	1.6	1.57	0.0088

Supplementary Table 5. **Top hits in proteomic analysis of RPE tissue from PF-9283-treated rats**. Analysis was performed on tryptic digests of RPE tissue prepared from rats dosed daily for 14 days with 80 mg kg^{-1} PF-9283. Peptide spectral matches (PSM) of the above 11 identified proteins (of 391 total identified proteins) were changed more than 1.5-fold ($P < 0.01$) compared to vehicle controls. Average PSM were calculated from 6 LCMS runs consisting of 2 technical replicates of $n = 3$ RPE tissues per group, and limited to those proteins with at least 5 average PSM in both treated and control samples. Significant differences were determined using a two-tailed *t*-test.

	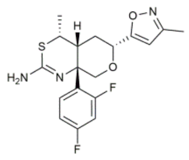	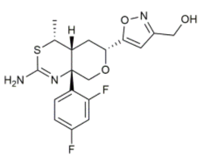	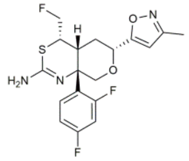	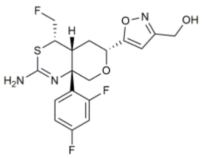	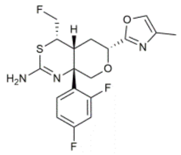	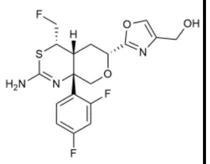
	**PF-9283 (4)**	**PF-9159 (11)**	**PF-3195 (9)**	**PF-3763 (12)**	**PF-1283 (10)**	**PF-7152 (13)**
CatD cellular IC_{50}	140 nM	110 nM	540 nM	940 nM	240 nM	580 nM
CatD enzyme IC_{50}	12 µM	17 µM	9.0 µM	16 µM	13 µM	37 µM
CatD potency increase in live cells	**86x**	**150x**	**17x**	**17x**	**54x**	**64x**
BACE1 cellular IC_{50}	6.0 nM	17 nM	15 nM	80 nM	10 nM	64 nM
BACE1 enzyme IC_{50}	74 nM	210 nM	62 nM	380 nM	120 nM	460 nM
BACE1 potency increase in live cells	**12x**	**12x**	**4.1x**	**4.8x**	**12x**	**7.2x**
BACE2 enzyme IC_{50}	140 nM	NA	150 nM	1.6 µM	95 nM	670 nM
CatD/BACE1 enzyme IC_{50}	**160**	**81**	**150**	**42**	**110**	**80**
CatD/BACE1 cellular IC_{50}	**23**	**6.5**	**36**	**12**	**24**	**9.1**
pKa	7.7	7.8	7.0	7.0	7.0	6.9

Supplementary Table 6. Comparison of aspartyl protease inhibitory activities of BACE1 inhibitors and metabolites. Enzyme IC_{50} values were determined using fluorescence polarization assays with purified human enzymes and fluorescent peptide substrates. BACE1 cellular IC_{50} values were determined by ELISA detection of sAPP-beta levels in H4 neuroglioma cells transfected with human wild-type APP. CatD cellular IC_{50} values were determined using the PF-7802 photoprobe in ARPE-19 cells. pK_a values were determined using a capillary electrophoresis method. NA: not available. Data are presented as the mean of at least three independent experiments.

Compound	Species	Duration (days)	Dose (mg kg^{-1})	Avg $C_{p,u}$ (nM) parent/metabolite	Male ocular toxicity	Female ocular toxicity
LY2811376	rat	7	25	741/NA	Pos 1/4	a
LY2811376	rat	7	75	4370/NA	Pos 4/4	a
PF-9283	rat	14	30	29/1490	Neg 3/3	Pos 1/3
PF-9283	dog	14	3	32/155	Neg 1/1	Neg 1/1
PF-9283	dog	14	9	401/2210	Pos 1/1	Neg 1/1
PF-3195	rat	14	15	5.4/460	Neg 3/3	Neg 3/3
PF-3195	rat	14	40	58/2570	Neg 3/3	Neg 3/3
PF-3195	rat	14	90	232/6350	Pos 1/3	Pos 2/3
PF-3195	rat	14	200	1790/17300	Pos 3/3	Pos 2/3
PF-1283	rat	14	45	109/352	Neg 3/3	Neg 3/3
PF-1283	rat	14	110	793/859	Neg 3/3	Pos 1/3
SG1	rat	29	60	1560/NA	Neg 4/4	Neg 4/4
LY2886721	rat	29	100	3480/NA	Neg 4/4	Neg 4/4

Supplementary Table 7. Summary of ocular findings. Outcomes were considered positive (pos) if at least one animal had accumulated autofluorescent granules in the RPE cytoplasm as determined by fluorescence microscopy and negative (neg) if there was no ocular abnormality observed. Entries are listed as number of positive or negative animals / total number of animals evaluated. Dosing was performed once daily. Avg $C_{p,u}$: average unbound plasma concentration in cohort. Sample sizes for plasma exposures and ocular toxicity determinations as indicated: LY2811376 - Rat 4 males, PF-9283 - Rat 3 males and 3 females, Dog 1 male and 1 female, PF-3195 - Rat 3 males and 3 females, PF1283 - Rat 3 males and 3 females, SG1 - Rat 4 males and 4 females, LY2886721 - Rat 4 males and 4 females. a: only males in study. NA: not applicable, no major metabolites.

Supplementary Methods

General synthetic methods. All commercially available reagents and solvents were used as received. Reactions were monitored by thin layer chromatography (TLC) performed on Analtech, Inc. silica gel GF 250 µm plates and were visualized with ultraviolet (UV) light (254 nm), $KMnO_4$ and/or ninhydrin staining or by UPLC-MS [Waters Acquity, ESCI (ESI +/-, APCI +/-)]. Silica gel flash chromatography was performed with RediSep Rf or Biotage disposable normal phase silica gel flash columns on a CombiFlash Rf system from Teledyne Isco, Inc. or a Biotage Horizon automatic purification system. Proton and carbon nuclear magnetic resonance (^{1}H NMR and ^{13}C NMR) spectra were recorded on a Varian-Inova 400 (400 MHz and 101 MHz, respectively), a Varian-Inova 600 (600 MHz and 151 MHz, respectively), a Bruker 400 (400 MHz and 101 MHz, respectively), or a Bruker 500 (500 MHz and 126 MHz, respectively) spectrometer. Chemical shifts are reported in ppm relative to $CHCl_3$ or MeOH (i.e. ^{1}H NMR δ = 7.26 and ^{13}C NMR δ = 77.0 or ^{1}H NMR δ = 3.31 and ^{13}C NMR δ = 49.1, respectively). The peak shapes are denoted as follows: s, singlet; d, doublet; t, triplet; q, quartet; m, multiplet; br., broad. High-resolution mass spectra (HRMS) were acquired on an Agilent model 6220 time-of-flight (TOF) mass spectrometer in positive or negative electrospray ionization (ESI) mode.

Compounds **15**, **18** and **23** were synthesized as previously described[1].

Synthesis of photoaffinity probe PF-7802 (6).

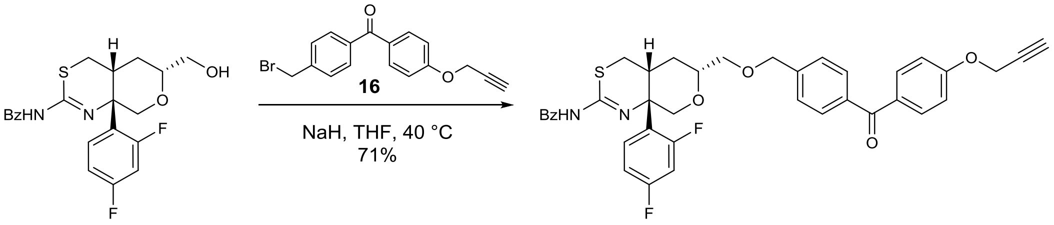

15 **17**

***N*-{(4a*R*,6*R*,8a*S*)-8a-(2,4-Difluorophenyl)-6-[({4-[4-(prop-2-yn-1-yloxy)benzoyl]benzyl}oxy)methyl]-4,4a,5,6,8,8a-hexahydropyrano[3,4-*d*][1,3]thiazin-2-yl}benzamide (17)**. To a vigorously stirring solution of **15** (465 mg, 1.11 mmol) in THF (4 mL) at room temperature was added sodium hydride (135 mg, 3.38 mmol, 60% dispersion in mineral oil) in portions. The mixture was stirred at room temperature for 30 minutes. Next, **16** (370 mg, 1.12 mmol) was added in one portion and the mixture was stirred at room temperature for 1 hour, then heated to 40 °C for 1.5 hours. The reaction mixture was cooled to room temperature, quenched with aqueous $KHSO_4$ solution (0.5 M, 20 mL), and extracted with EtOAc (3x). The combined organic extracts were washed with brine (1x), dried with anhydrous Na_2SO_4, filtered, and concentrated under reduced pressure. The crude residue was purified by silica gel chromatography (EtOAc/heptane) to afford **17** (525 mg, 71% yield) as a white solid. ^{1}H NMR (400 MHz, $CDCl_3$) δ = 11.99 (br. s, 1H), 8.27 - 8.13 (m, 2H), 7.85 - 7.75 (m, 2H), 7.69 (d, *J* = 8.2 Hz, 2H), 7.54 - 7.35 (m, 6H), 7.08 - 6.98 (m, 2H), 6.97 - 6.82 (m, 2H), 4.83 - 4.74 (m, 2H), 4.72 - 4.58 (m, 2H), 4.22 - 4.14 (m, 1H), 4.00 - 3.91 (m, 1H), 3.84 (d, *J* = 12.5 Hz, 1H), 3.68 (dd, *J* = 6.2, 10.1 Hz, 1H), 3.60 - 3.51 (m, 1H), 3.24 - 3.10 (m, 1H), 3.01 (dd, *J* = 4.3, 12.9 Hz, 1H), 2.65 (dd, *J* =

2.7, 12.9 Hz, 1H), 2.56 (t, J = 2.3 Hz, 1H), 2.10 - 1.97 (m, 1H), 1.75 - 1.66 (m, 1H). ^{13}C NMR (101 MHz, $CDCl_3$) δ = 195.10, 163.02 (dd, $^{1}J_{CF}$ = 251.53 Hz, $^{3}J_{CF}$ = 12.32 Hz), 160.93, 158.83 (dd, $^{1}J_{CF}$ = 247.95 Hz, $^{3}J_{CF}$ = 11.52 Hz), 142.30, 137.35, 136.40, 132.36, 132.01, 130.98, 130.93 (dd, $^{3}J_{CF}$ = 9.54 Hz, $^{3}J_{CF}$ = 5.56 Hz), 129.96, 129.27, 128.12, 127.20, 114.38, 112.33 (dd, $^{2}J_{CF}$ = 20.66 Hz, $^{4}J_{CF}$ = 3.18 Hz), 105.6 (dd, $^{2}J_{CF}$ = 28.61 Hz, $^{2}J_{CF}$ = 25.43 Hz), 77.80, 77.21, 76.59, 76.14, 73.11, 73.08, 59.33 (d, $^{3}J_{CF}$ = 6.76 Hz), 55.86, 31.38 (d, $^{4}J_{CF}$ = 5.96 Hz), 29.41, 27.64. HRMS calculated for $C_{38}H_{32}F_2N_2NaO_5S$ $[M+Na]^+$ 689.1892, found 689.1891.

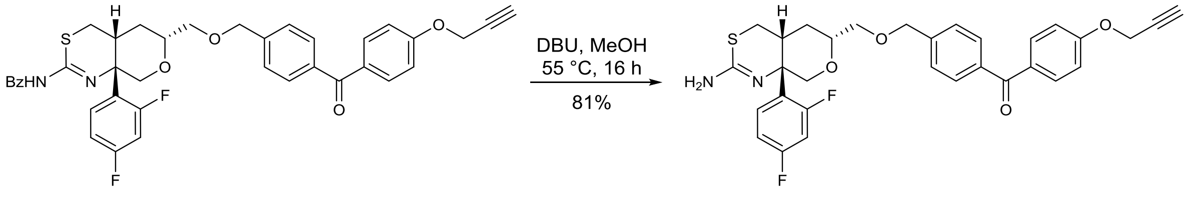

17 **6**

[4-({[(4a*R*,6*R*,8a*S*)-2-amino-8a-(2,4-difluorophenyl)-4,4a,5,6,8,8a-hexahydropyrano[3,4-*d*][1,3]thiazin-6-yl]methoxy}methyl)phenyl][4-(prop-2-yn-1-yloxy)phenyl]methanone (6, PF-7802). To a solution of **17** (300 mg, 0.45 mmol) in methanol (4.5 mL) was added DBU (45.0 µL, 45.8 mg, 0.301 mmol). The reaction mixture was heated at 55 °C in a sealed vial for 16 hours. Water was added and the mixture was extracted with EtOAc (3x). The combined organic extracts were dried over anhydrous Na_2SO_4, filtered, and concentrated under reduced pressure. The crude residue was purified by silica gel chromatography ($MeOH/CH_2Cl_2$) to afford **6** (206 mg, 81% yield) as a white solid. ^{1}H NMR (400 MHz, $CDCl_3$) δ = 7.87 - 7.79 (m, 2H), 7.79 - 7.68 (m, 2H), 7.45 (d, J = 8.2 Hz, 2H), 7.36 (dt, J = 6.6, 9.0 Hz, 1H), 7.08 - 7.01 (m, 2H), 6.89 (dt, J = 2.3, 8.2 Hz, 1H), 6.80 (ddd, J = 2.3, 8.7, 12.4 Hz, 1H), 5.68 (br. s, 2H), 4.81 - 4.76 (m, 2H), 4.72 - 4.62 (m, 2H), 4.09 (dd, J = 2.0, 11.3 Hz, 1H), 3.95 - 3.83 (m, 2H), 3.66 (dd, J = 6.2, 10.1 Hz, 1H), 3.53 (dd, J = 4.5, 10.3 Hz, 1H), 3.02 - 2.91 (m, 2H), 2.66 - 2.59 (m, 1H), 2.57 (t, J = 2.3 Hz, 1H), 1.89 - 1.75 (m, 1H), 1.62 - 1.49 (m, 1H). ^{13}C NMR (101 MHz, $CDCl_3$) δ = 195.20, 162.49 (dd, $^{1}J_{CF}$ = 250.15 Hz, $^{3}J_{CF}$ = 12.47 Hz), 160.98, 160.95, 159.08 (dd, $^{1}J_{CF}$ = 247.96 Hz, $^{3}J_{CF}$ = 11.74 Hz), 142.54, 137.34, 132.41, 131.96 (dd, $^{3}J_{CF}$ = 9.54 Hz, $^{3}J_{CF}$ = 5.87 Hz), 130.97, 130.00, 129.95, 127.27, 114.41, 111.76 (dd, $^{2}J_{CF}$ = 20.54 Hz, $^{4}J_{CF}$ = 2.93 Hz), 105.00 (dd, $^{2}J_{CF}$ = 28.61 Hz, $^{2}J_{CF}$ = 25.68 Hz), 77.77, 76.35, 76.16, 75.71 (d, $^{4}J_{CF}$ = 4.77 Hz), 73.34, 72.99, 58.61 (d, $^{3}J_{CF}$ = 6.60 Hz), 55.86, 29.16 (d, $^{4}J_{CF}$ = 6.60 Hz), 29.02, 28.02. HRMS calculated for $C_{31}H_{29}F_2N_2O_4S$ $[M+H]^+$ 563.1811, found 563.1819.

Synthesis of metabolite PF-3763 (12).

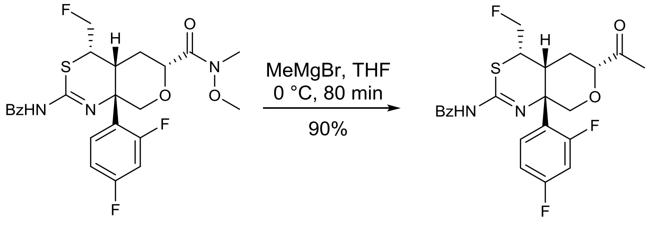

18 **19**

N-((4S,4aR,6R,8aS)-6-acetyl-8a-(2,4-difluorophenyl)-4-(fluoromethyl)-4,4a,5,6,8,8a-hexahydropyrano[3,4-d][1,3]thiazin-2-yl)benzamide (19). To a solution of **18** (495 mg, 0.975 mmol) in THF (10 mL) at 0 °C was added methylmagnesium bromide (0.975 mL, 2.92 mmol, 3.0 M in THF) over a period of 6 minutes. The mixture was stirred at 0 °C for 80 minutes and then quenched by the careful addition of saturated aqueous NH_4Cl solution (20 mL). The mixture was extracted with EtOAc (3x) and the combined organic extracts were dried with anhydrous Na_2SO_4, filtered, and concentrated under reduced pressure. The crude residue was purified by silica gel chromatography (EtOAc/heptane) to afford **19** (406 mg, 90% yield) as a white solid. ^{1}H NMR (400 MHz, $CDCl_3$) δ = 12.28 (br. s, 1H), 8.25 - 8.12 (m, 2H), 7.58 - 7.51 (m, 1H), 7.51 - 7.43 (m, 2H), 7.41 - 7.33 (m, 1H), 7.01 - 6.87 (m, 2H), 4.68 - 4.34 (m, 2H), 4.21 (d, *J* = 12.5 Hz, 1H), 4.08 - 4.02 (m, 1H), 3.93 (d, *J* = 12.1 Hz, 1H), 3.57 - 3.47 (m, 1H), 3.30 - 3.19 (m, 1H), 2.26 (s, 3H), 2.04 - 1.97 (m, 1H), 1.84 - 1.72 (m, 1H). LRMS *m/z*: 463.2 [M+H]$^+$.

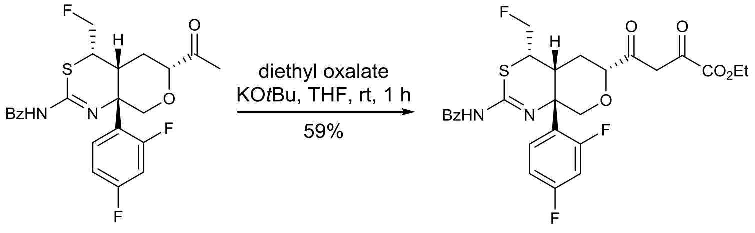

19 **20**

ethyl 4-((4S,4aR,6R,8aS)-2-benzamido-8a-(2,4-difluorophenyl)-4-(fluoromethyl)-4,4a,5,6,8,8a-hexahydropyrano[3,4-d][1,3]thiazin-6-yl)-2,4-dioxobutanoate (20). To a solution of potassium *tert*-butoxide (260 mg, 2.27 mmol) in THF (7.6 mL) at 0 °C was added **19** (350 mg, 0.76 mmol) and diethyl oxalate (332 mg, 2.27 mmol). The reaction mixture was stirred at room temperature for 1 hour and then poured into saturated aqueous NH_4Cl solution. The mixture was extracted with EtOAc (3x) and the combined organic extracts were dried with anhydrous Na_2SO_4, filtered, and concentrated under reduced pressure. The crude residue was purified by silica gel chromatography (EtOAc/heptane) to afford **20** (250 mg, 59% yield) as a white solid. LRMS *m/z*: 563.2 [M+H]$^+$.

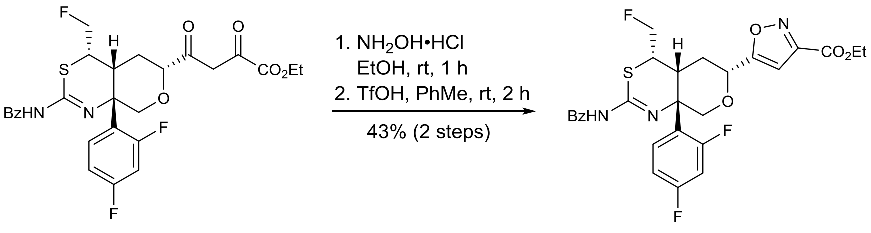

20 **21**

Ethyl 5-((4S,4aR,6R,8aS)-2-benzamido-8a-(2,4-difluorophenyl)-4-(fluoromethyl)-4,4a,5,6,8,8a-hexahydropyrano[3,4-d][1,3]thiazin-6-yl)isoxazole-3-carboxylate (21). To a solution of **20** (250 mg, 0.44 mmol) in EtOH (4.4 mL) was added hydroxylamine hydrochloride (33.9 mg, 0.488 mmol) in one portion. The reaction mixture was stirred at room temperature for 1 hour, at which point LCMS showed desired product. The reaction mixture was concentrated under reduced pressure. The crude residue was dissolved in toluene (4.2 mL), and

trifluoromethanesulfonic acid (562 µL, 6.36 mmol) was added in one portion. The reaction was stirred at room temperature for 2 hours. The mixture was neutralized with 1 N aqueous NaOH solution and extracted with EtOAc (3x). The combined organic extracts were dried with anhydrous Na_2SO_4, filtered, and concentrated under reduced pressure. The crude residue was purified by silica gel chromatography (EtOAc/heptane) to afford **21** (102 mg, 43% yield) as a white solid. LRMS *m/z*: 560.3 $[M+H]^+$.

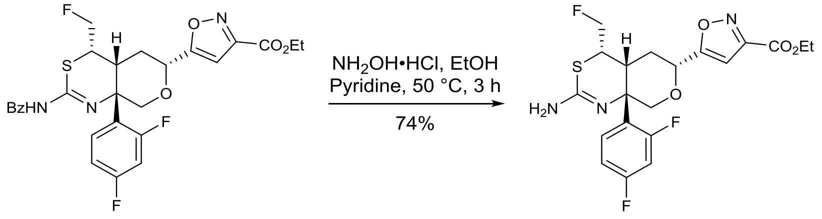

21 **22**

ethyl 5-((4S,4aR,6R,8aS)-2-amino-8a-(2,4-difluorophenyl)-4-(fluoromethyl)-4,4a,5,6,8,8a-hexahydropyrano[3,4-d][1,3]thiazin-6-yl)isoxazole-3-carboxylate (22). A solution of **21** (53 mg, 95 µmol) and hydroxylamine hydrochloride (79 mg, 0.95 mmol) in ethanol (5 mL) was treated with pyridine (770 µL, 9.47 mmol) and then heated to 50 °C for 3 h. LCMS indicated starting material had been consumed and a new product had formed. The reaction mixture was concentrated to ~1/2 volume, then taken up in CH_2Cl_2 and washed with 1 N aqueous NaOH solution. The aqueous phase was extracted with CH_2Cl_2 (2x). The combined organic extracts were washed with water (1x) and brine (1x), dried with anhydrous Na_2SO_4, and concentrated under reduced pressure. The crude residue was purified by silica gel chromatography ($MeOH/CH_2Cl_2$) to afford **22** (32 mg, 74% yield) as a white solid. ^1H NMR (500 MHz, $CDCl_3$) δ = 7.34 (dt, *J* = 6.6, 9.0 Hz, 1H), 6.93 - 6.81 (m, 2H), 6.73 (s, 1H), 4.92 - 4.86 (m, 1H), 4.80 - 4.55 (m, 3H), 4.54 - 4.31 (m, 3H), 4.26 (dd, *J* = 2.0, 11.2 Hz, 1H), 3.95 (d, *J* = 11.0 Hz, 1H), 3.57 - 3.48 (m, 1H), 3.19 - 3.12 (m, 1H), 1.98 - 1.92 (m, 2H), 1.41 (t, *J* = 7.3 Hz, 3H). LRMS *m/z*: 456.1 $[M+H]^+$.

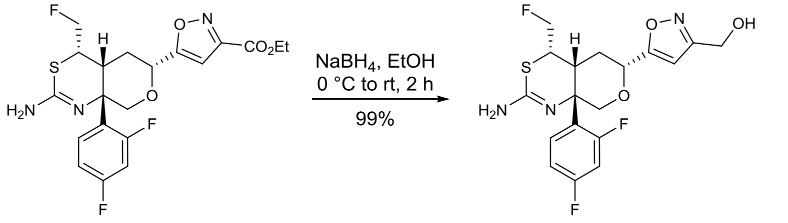

22 **12**

(5-((4S,4aR,6R,8aS)-2-amino-8a-(2,4-difluorophenyl)-4-(fluoromethyl)-4,4a,5,6,8,8a-hexahydropyrano[3,4-d][1,3]thiazin-6-yl)isoxazol-3-yl)methanol (12, PF-3763). A solution of **22** (11.1 mg, 24.4 µmol) in EtOH (1.5 mL) was cooled to 0 °C. Sodium borohydride (2.7 mg, 73 µmol) was then added in one portion. The ice bath was removed and the mixture was stirred at room temperature for 2 hours, after which LCMS indicated consumption of starting material. EtOAc was added and the mixture was washed with saturated aqueous NH_4Cl solution (2x) and brine (1x). The organic extract was dried with anhydrous $MgSO_4$, filtered, and concentrated under reduced pressure. The crude residue was purified by silica gel chromatography (2 N NH_3 in

MeOH/EtOAc) to afford **12** (10 mg, 99% yield) as a white solid. ^{1}H NMR (500 MHz, CD_3OD) δ = 7.38 (dt, *J* = 6.6, 8.8 Hz, 1H), 7.05 - 6.95 (m, 2H), 6.41 (s, 1H), 4.91 - 4.87 (m, 1H), 4.71 - 4.56 (m, 3H), 4.50 - 4.35 (m, 1H), 4.29 (dd, *J* = 2.0, 11.2 Hz, 1H), 3.83 (d, *J* = 11.2 Hz, 1H), 3.43 (dtd, *J* = 3.7, 6.8, 13.8 Hz, 1H), 3.22 - 3.15 (m, 1H), 1.98 - 1.88 (m, 2H). ^{13}C NMR (126 MHz, CD_3OD) δ = 173.52, 165.56, 164.32 (dd, $^1J_{CF}$ = 249.08 Hz, $^3J_{CF}$ = 12.62 Hz), 161.00 (dd, $^1J_{CF}$ = 247.39 Hz, $^3J_{CF}$ = 11.78 Hz), 157.37, 133.44 (dd, $^3J_{CF}$ = 9.25 Hz, $^3J_{CF}$ = 5.89 Hz), 125.82 (dd, $^2J_{CF}$ = 12.62 Hz, $^4J_{CF}$ = 3.37 Hz), 112.74 (dd, $^2J_{CF}$ = 20.20 Hz, $^4J_{CF}$ = 3.37 Hz), 106.17 (dd, $^2J_{CF}$ = 28.61 Hz, $^2J_{CF}$ = 26.09 Hz), 101.51, 84.52 (d, $^1J_{CF}$ = 172.50 Hz), 76.80 (d, $^4J_{CF}$ = 5.05 Hz), 72.77, 61.27 (d, $^3J_{CF}$ = 5.89 Hz), 56.88, 42.29 (d, $^2J_{CF}$ = 20.20 Hz), 33.00 (dd, $^3J_{CF}$ = 6.73 Hz, $^4J_{CF}$ = 5.05 Hz), 27.22. HRMS calculated for $C_{18}H_{19}F_3N_3O_3S$ $[M+H]^+$ 414.1094, found 414.1088.

Synthesis of metabolite PF-7152 (13).

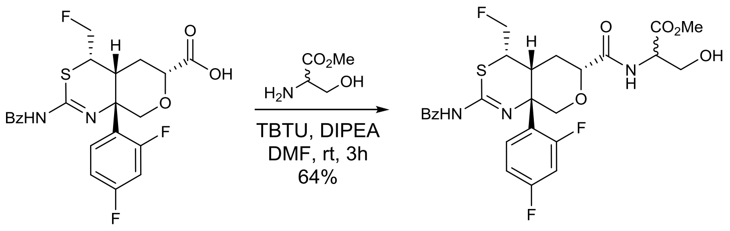

23 24

methyl((4S,4aR,6R,8aS)-2-benzamido-8a-(2,4-difluorophenyl)-4-(fluoromethyl)-4,4a,5,6,8,8a-hexahydropyrano[3,4-d][1,3]thiazine-6-carbonyl)serinate (24). To a solution of **23** (1.05 g, 2.26 mmol) in DMF (7.5 mL) was added DIPEA (2.36 mL, 13.6 mmol) followed by TBTU (738 mg, 2.49 mmol) in one portion. The mixture was stirred at room temperature for 35 minutes and then DL-serine methyl ester hydrochloride (1.23 g, 7.91 mmol) was added in one portion and the reaction was stirred at room temperature for 3 hours. The reaction mixture was diluted with saturated aqueous $NaHCO_3$ solution (30 mL) and water (30 mL) and extracted with MTBE (3x). The combined organic extracts were dried with anhydrous Na_2SO_4, filtered, and concentrated under reduced pressure. The crude residue was purified by silica gel chromatography (EtOAc/heptane) to afford **24** (820 mg, 64% yield, mixture of diastereomers) as a solid. ^{1}H NMR (400 MHz, $CDCl_3$) δ = 8.15 (d, *J* = 7.4 Hz, 2H), 7.59 - 7.52 (m, 1H), 7.50 - 7.42 (m, 3H), 7.39 - 7.31 (m, 1H), 6.99 - 6.86 (m, 2H), 4.72 - 4.34 (m, 3H), 4.27 - 4.16 (m, 2H), 4.04 - 3.91 (m, 2H), 3.84 (dd, *J* = 3.1, 11.3 Hz, 1H), 3.80 - 3.65 (m, 3H), 3.57 - 3.46 (m, 1H), 3.31 - 3.20 (m, 1H), 2.29 - 2.17 (m, 1H), 1.89 - 1.76 (m, 1H), 1.70 (br. s, 2H) (mixture of diastereomers). LRMS *m/z*: 566.3 $[M+H]^+$.

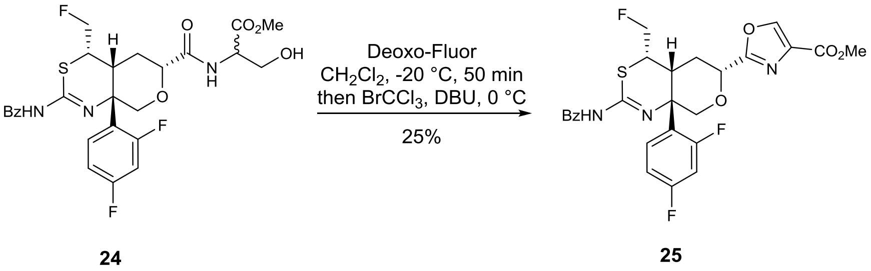

methyl 2-((4S,4aR,6R,8aS)-2-benzamido-8a-(2,4-difluorophenyl)-4-(fluoromethyl)-4,4a,5,6,8,8a-hexahydropyrano[3,4-d][1,3]thiazin-6-yl)oxazole-4-carboxylate (25). To a solution of **24** (820 mg, 1.45 mmol) in CH_2Cl_2 (29 mL) at −20 °C was added Deoxo-Fluor (417 mg, 1.88 mmol) and the mixture was stirred at −20 °C for 50 minutes. Bromotrichloromethane (0.53 mL, 5.4 mmol) and DBU (0.84 mL, 5.4 mmol) were added and the mixture was then warmed to 0 °C and stirred overnight. The mixture was diluted with saturated aqueous $NaHCO_3$ solution and extracted with CH_2Cl_2 (3x). The combined organic extracts were dried with anhydrous Na_2SO_4, filtered, and concentrated under reduced pressure. The crude residue was purified by silica gel chromatography (EtOAc/heptane) to afford **25** (200 mg, 25% yield) as a white solid. ^{1}H NMR (400 MHz, $CDCl_3$) δ = 8.23 (s, 1H), 8.12 (d, *J* = 7.4 Hz, 2H), 7.57 - 7.50 (m, 1H), 7.49 - 7.42 (m, 2H), 7.42 - 7.31 (m, 1H), 6.98 - 6.85 (m, 2H), 4.90 (dd, *J* = 2.3, 11.7 Hz, 1H), 4.71 - 4.36 (m, 2H), 4.33 (d, *J* = 10.9 Hz, 1H), 3.98 (d, *J* = 12.1 Hz, 1H), 3.90 (s, 3H), 3.59 - 3.49 (m, 1H), 3.39 - 3.31 (m, 1H), 2.40 - 2.28 (m, 1H), 2.22 - 2.14 (m, 1H). LRMS *m/z*: 546.3 $[M+H]^+$.

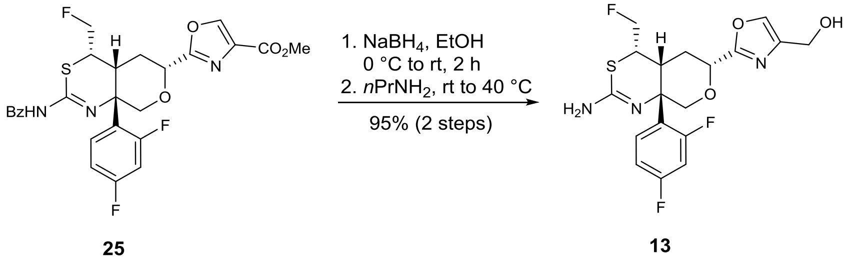

(2-((4S,4aR,6R,8aS)-2-amino-8a-(2,4-difluorophenyl)-4-(fluoromethyl)-4,4a,5,6,8,8a-hexahydropyrano[3,4-d][1,3]thiazin-6-yl)oxazol-4-yl)methanol (13, PF-7152). A solution of **25** (36 mg, 66 μmol) in EtOH (1.5 mL) was cooled to 0 °C. Sodium borohydride (7.5 mg, 0.20 mmol) was then added in one portion. The ice bath was removed and the mixture was stirred at room temperature for 2 hours, after which LCMS indicated consumption of starting material. EtOAc was added and the mixture was washed with saturated aqueous NH_4Cl solution (2x) and brine (1x). The organic layer was dried with anhydrous $MgSO_4$, filtered, and concentrated under reduced pressure. To the crude solid was added propylamine (1 mL) and the mixture was stirred at room temperature overnight, then heated to 40 °C for 1 hour, after which LCMS indicated consumption of starting material. The mixture was then concentrated under reduced pressure. The crude reside was purified by silica gel chromatography (2N NH_3 in MeOH/EtOAc) to afford **13** (25 mg, 92% yield over 2 steps) as a white solid. ^{1}H NMR (400 MHz, CD_3OD) δ = 7.80 (s, 1H), 7.43 - 7.29 (m, 1H), 7.07 - 6.94 (m, 2H), 4.82 (dd, *J* = 2.5, 11.9 Hz, 1H), 4.73 - 4.55 (m, 1H), 4.51 (d, *J* = 0.8 Hz, 2H), 4.50 - 4.34 (m, 1H), 4.28 (dd, *J* = 2.0, 10.9 Hz, 1H), 3.82 (d, *J* = 11.3 Hz, 1H), 3.43 (dtd, *J* = 3.7, 6.7, 13.7 Hz, 1H), 3.17 (td, *J* = 3.9, 12.1 Hz, 1H), 2.16 (dq, *J* = 2.0, 12.6 Hz, 1H), 1.89 (td, *J*

= 3.3, 13.0 Hz, 1H). ^{13}C NMR (100 MHz, CD_3OD) δ = 164.34, 164.32 (dd, $^1J_{CF}$ = 248.69 Hz, $^3J_{CF}$ = 13.20 Hz), 160.70 (dd, $^1J_{CF}$ = 277.22 Hz, $^3J_{CF}$ = 11.74 Hz), 157.23, 142.23, 137.77, 133.48 (dd, $^3J_{CF}$ = 9.54 Hz, $^3J_{CF}$ = 5.87 Hz), 125.85 (dd, $^2J_{CF}$ = 12.47 Hz, $^4J_{CF}$ = 3.67 Hz), 112.73 (dd, $^2J_{CF}$ = 21.27 Hz, $^4J_{CF}$ = 3.30 Hz), 106.17 (dd, $^2J_{CF}$ = 25.68 Hz, $^2J_{CF}$ = 29.34 Hz), 84.55 (d, $^1J_{CF}$ = 172.39), 77.35 (d, $^4J_{CF}$ = 4.40 Hz), 73.24, 60.24 (d, $^3J_{CF}$ = 6.60 Hz), 57.14, 42.31 (d, $^2J_{CF}$ = 19.81 Hz), 32.87 (app t, $^3J_{CF}$ = 5.51 Hz, $^4J_{CF}$ = 5.51 Hz), 26.05. HRMS calculated for $C_{18}H_{19}F_3N_3O_3S$ $[M+H]^+$ 414.1094, found 414.1091.

Synthesis of Metabolite PF-2696 (14).

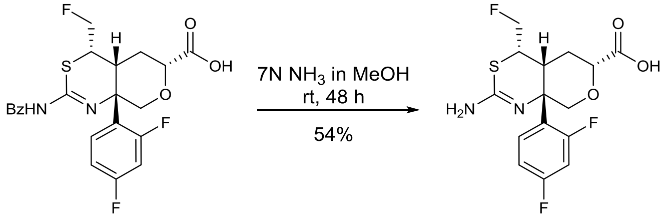

23 **14**

(4S,4aR,6R,8aS)-2-amino-8a-(2,4-difluorophenyl)-4-(fluoromethyl)-4,4a,5,6,8,8a-hexahydropyrano[3,4-d][1,3]thiazine-6-carboxylic acid (14, PF-2696). A solution of **23** (0.100 g, 0.215 mmol) in 7 N ammonia in methanol (2.15 mL) was stirred at room temperature for 48 hours, after which LCMS indicated consumption of starting material. The mixture was concentrated under reduced pressure. The residue was dissolved in dimethyl sulfoxide (1 mL) and purified by reversed-phase HPLC (Column: Waters XBridge C18, 19 x 100 mm, 5 μm; Mobile phase A: 0.03% NH_4OH in water (v/v); Mobile phase B: 0.03% NH_4OH in acetonitrile (v/v); Gradient: 95.0% A/5.0% B linear to 60% A/40% B in 8.5 min, linear to 0% A/100% B to 9.0 min, HOLD at 0% A/100% B to 10.0 min. Flow: 25 mL/min. Collection by mass at 361.08 *m/z* afforded **14** (44 mg, 54% yield, ammonium salt) as a white solid. ^1H NMR (500 MHz, CD_3OD) δ = 7.46 - 7.35 (m, 1H), 7.16 - 7.01 (m, 2H), 4.83 - 4.69 (m, 1H), 4.66 - 4.49 (m, 1H), 4.07 (dd, J = 2.4, 12.0 Hz, 2H), 3.96 (d, J = 12.2 Hz, 1H), 3.65 (br. s, 1H), 3.39 (td, J = 3.9, 12.0 Hz, 1H), 2.19 - 2.04 (m, 1H), 1.83 - 1.63 (m, 1H). ^{13}C NMR (126 MHz, CD_3OD) δ = 178.84, 168.31, 164.90 (dd, $^1J_{CF}$ = 250.76 Hz, $^3J_{CF}$ = 12.62 Hz), 161.02 (br. d, $^1J_{CF}$ = 250.76Hz), 131.87 (br. s), 123.16 (dd, $^2J_{CF}$ = 10.94 Hz, $^4J_{CF}$ = 3.37 Hz), 113.62 (dd, $^2J_{CF}$ = 21.04 Hz, $^4J_{CF}$ = 3.36 Hz), 106.98 (dd, $^2J_{CF}$ = 28.61 Hz, $^2J_{CF}$ = 26.09 Hz), 84.06 (d, $^1J_{CF}$ = 173.34 Hz), 78.52, 73.14 (br. s), 61.19 (br. s), 43.55 (d, $^2J_{CF}$ = 19.35 Hz), 35.84 (app t, $^3J_{CF}$ = 5.47 Hz, $^4J_{CF}$ = 5.47 Hz), 26.33. HRMS calculated for $C_{15}H_{16}F_3N_2O_3S$ $[M+H]^+$ 361.0828, found 361.0823.

Supplementary References

1 Brodney, M.A. *et al.* Utilizing CYP2D6 and BACE1 Structure complexes to reduce risk of drug-drug interactions with a novel series of centrally efficacious BACE1 inhibitors. *J. Med. Chem.* **58**, 3223-3252 (2015).

2 May, P.C. *et al.* Robust central reduction of Amyloid-β in humans with an orally available, non-peptidic β-secretase inhibitor. *J. Neurosci.* **31**, 16507-16516 (2011).

3 Dineen, T.A. *et al.* Inhibitors of β-site amyloid precursor protein cleaving enzyme (BACE1): identification of (S)-7-(2-fluoropyridin-3-yl)-3-((3-methyloxetan-3-yl)ethynyl)-5′H-spiro[chromeno[2,3-b]pyridine-5,4′-oxazol]-2′-amine (AMG-8718). *J. Med. Chem.* **57**, 9811-9831 (2014).

4 Stachel, S.J. *et al.* Structure-based design of potent and selective cell-permeable inhibitors of human β-secretase (BACE-1). *J. Med. Chem.* **47**, 6447-6450 (2004).

5 May, P.C. *et al.* The potent BACE1 inhibitor LY2886721 elicits robust central Aβ pharmacodynamic responses in mice, dogs, and humans. *J. Neurosci.* **35**, 1199-1210 (2015).
